# Social Understanding beyond the Familiar: Disparity in Visual Abilities Does Not Impede Empathy and Theory of Mind

**DOI:** 10.3390/jintelligence12010002

**Published:** 2023-12-25

**Authors:** Eva Landmann, Alina Krahmer, Anne Böckler

**Affiliations:** Department of Psychology, University of Würzburg, 97070 Würzburg, Germanyanne.boeckler@uni-wuerzburg.de (A.B.)

**Keywords:** empathy, theory of mind, social understanding, communication, visual impairment, sharing perceptual experience

## Abstract

Feeling with our conspecifics and understanding their sentiments and intentions is a crucial part of our lives. What is the basis for these forms of social understanding? If individuals ground their understanding of others’ thoughts and feelings in their own perceptual and factual experiences, it could present a challenge to empathize and mentalize with those whose reality of life is significantly different. This preregistered study compared two groups of participants who differed in a central perceptual feature, their visual abilities (visually impaired vs. unimpaired; total N = 56), concerning their social understanding of others who were themselves either visually impaired or unimpaired. Employing an adjusted version of the EmpaToM task, participants heard short, autobiographic narrations by visually impaired or unimpaired individuals, and we assessed their empathic responding and mentalizing performance. Our findings did not reveal heightened empathy and mentalizing proclivities when the narrator’s visual abilities aligned with those of the participant. However, in some circumstances, cognitive understanding of others’ narrations benefitted from familiarity with the situation. Overall, our findings suggest that social understanding does not mainly rely on perceptual familiarity with concrete situations but is likely grounded in sharing emotions and experiences on a more fundamental level.

## 1. Introduction

There can be significant differences in the realities of life between any two individuals. Even those living door-to-door will have markedly different experiences in their surroundings. For example, when walking to the bus stop, most residents would naturally notice street signs, church steeples, or brightly painted houses they pass on their way. However, if one of the neighbors happened to be visually impaired, they would probably experience the same route through the texture of the pavement, the babbling of a fountain, and the smell of bread from the bakery. As a result, the mental representations these individuals form of their neighborhood presumably differ. After all, our cognitive representations are not isolated from our sensory and motor systems but are, on the contrary, innately ‘grounded’ in perceptual experiences (Barsalou 2010; Kiefer and Barsalou 2013; Pecher and Zwaan 2005). Social cognition is also significantly influenced by our history of (inter)personal encounters and occurrences, which form the basis for our understanding of concepts like emotions, intentions, and beliefs (Bowlby 1973; Rokita et al. 2018; Vygotskij and Cole 1978). For instance, anyone who has ever been in a passive-aggressive argument will get the true message of a snarky ‘It’s nothing.’ when they overhear it in someone else’s quarrel and correctly infer the speaker’s dissatisfied emotional state. But what happens if one is trying to understand the perspective and feelings of another person whose reality of life and way of experiencing the environment distinctly differs from one’s own?

In the present study, we will focus on the impact of a similar vs. dissimilar reality of life on two central components of social understanding: empathy, which refers to mirroring another person’s feelings (De Vignemont and Singer 2006; Singer and Lamm 2009), and theory of mind (ToM), i.e., reasoning about other people’s mental states (beliefs, intentions, etc.; Frith and Frith 2005, 2006; Ho et al. 2022). These functions have been shown to be distinct in terms of neural networks, modulators, and the types of training they benefit from (Kanske et al. 2015; McDonald et al. 2022; Trautwein et al. 2020). Both, however, seem to be facilitated by similarities shared between the interaction partners: a number of studies report stronger empathic responses toward individuals belonging to the same ingroup, e.g., regarding ethnicity or political ideology (Gutsell and Inzlicht 2012; Neumann et al. 2013; Tarrant et al. 2009; Vanman 2016), as well as for individuals sharing similar values or internal conflicts (Heinke and Louis 2009; Nelson et al. 2003). Beyond personality and attitudes, some studies focused specifically on similarity in experiences and found heightened levels of sympathy and concern when participants had gone through similar life experiences as the person they encountered, for instance, giving birth to a child (Hodges et al. 2010), becoming a victim of sexual assault (Barnett et al. 1987), or suffering from teenage acne (Batson et al. 1996). Although research regarding a ’similarity benefit’ for ToM is not as extensive and conclusive, there are some indications that individuals find it easier to take another person’s perspective when they have had similar past experiences (Gerace et al. 2015) and that ToM performance is improved among individuals who belong to the same ethnic group or nationality (Gönültaş et al. 2020; Zhu et al. 2023). This pattern of results could be taken to suggest that people are particularly prone and capable of empathizing and mentalizing with those who share a similar reality of life.

On the other hand, humans’ social understanding would be severely limited if it depended on having the exact same experiences as their interaction partners. Most of us have probably, at one point or another, adopted the perspective of someone vastly different from ourselves (e.g., an alien character in a sci-fi movie). In line herewith, studies have reported participants taking the perspective of individuals who lived through situations that they themselves could never encounter, such as biologically female participants taking the perspective of a man suffering from testicular cancer (e.g., Van Boven and Loewenstein 2005). Likewise, people also empathize with members of an outgroup, with studies indicating a reduction or even absence of ingroup bias when relevant norms are activated (Tarrant et al. 2009) or when there was extensive prior contact with the relevant outgroup (Cao et al. 2015; Zuo and Han 2013). So, if—as these observations suggest—humans possess the necessary bases for empathy and ToM toward all kinds of others, to what extent does social understanding need to be grounded in concrete, corresponding experiences? For emotional states, the key might be to recognize immediate signals of a particular emotion in our interaction partner. If you come across a student crying outside the lecture hall, you probably feel for them despite not knowing the reason for their distress. And even upon learning that they just ripped their pants in front of the whole auditorium—something you were fortunate enough to never encounter yourself—you can probably draw on your own memories of running against doorframes or accidentally hitting ‘reply all’ on an email to imagine the wave of embarrassment and humiliation that would cause. In such instances, empathic resonance might be based on one’s own experience with the emotion rather than familiarity with the specific situation. But how about the simulation of more complex mental states, such as predicting the confusion of a person with severe hearing impairment when you dwell on the dangers of electric cars in pedestrian zones?

In order to effectively investigate and differentiate the potential grounding of social understanding, it is essential to study empathy and ToM not in isolation but within contextually rich settings. While many studies in the past have employed simplified and arbitrary stimuli, such as cartoon faces or isolated image details of the eye area, there has been increasing demands for the incorporation of more realistic and dynamic stimuli (Lehmann et al. 2019; Schilbach 2015; Schilbach et al. 2013) and the grounding of study designs in ecologically valid contexts (Osborne-Crowley 2020; Shamay-Tsoory and Mendelsohn 2019). In the present study, we employed naturalistic social stimuli that offered comprehensive contextual grounding to investigate the effect of similar vs. dissimilar realities of life. Specifically, we focused on a basic feature that profoundly influences how individuals perceive and experience their environment, namely, visual abilities. In our society, numerous facets of everyday life are inherently designed to accommodate individuals with unimpaired vision. This encompasses aspects such as mobility, occupational requirements, access to information, and social interactions. While visual cues play a pivotal role for the majority of the population, individuals with visual impairments undergo significantly different experiences in their lives and face specific challenges (Brown et al. 2014; Riazi et al. 2016). We therefore pose the following question: do individuals exhibit enhanced empathy and ToM performance toward others whose visual ability (impaired vs. unimpaired) matches their own? To investigate, we conducted an experiment on two groups of participants, one with and one without visual impairment, who listened to autobiographic narrations from others with and without visual impairments. We based our experimental design on the EmpaToM (Kanske et al. 2015), a validated paradigm measuring empathy and ToM performance. Rather than the original short video stimuli, we presented audio clips that featured individuals narrating brief episodes from their everyday lives. Crucially, the narrations were told from the perspective of either a person with visual impairment or without visual impairment. Subsequently, participants answered questions regarding their own emotional states and related to the content of the narrations, allowing us to calculate empathic resonance and ToM performance.

Considering the body of research reporting enhanced social understanding between individuals who share an ingroup or similar experiences (Batson et al. 1996; Gönültaş et al. 2020; Hodges et al. 2010; Tarrant et al. 2009; Vanman 2016; Zhu et al. 2023), one could anticipate comparable advantages (i.e., enhanced empathic responses and mentalizing performance) when the narrators’ visual abilities match those of the participants (Hypothesis A). On the other hand, processes of social understanding are flexible, and humans can feel for and comprehend the perspective of individuals who are vastly dissimilar to them (Cao et al. 2015; Van Boven and Loewenstein 2005; Zuo and Han 2013). Hence, irrespective of the (mis-)match in visual abilities, participants might accurately and equally understand the narrator’s perspective and empathize with them (Hypothesis B). We think that the results of the present study will also provide some information regarding the level at which grounding influences social understanding. Support for Hypothesis A, i.e., enhanced social affect and cognition when visual (dis)abilities are shared, would indicate that social understanding clearly benefits or even depends on the sharing of concrete experiences that are shaped by one’s reality of life. By contrast, support for Hypothesis B, i.e., unimpeded empathizing and mentalizing by different visual abilities, would suggest that social understanding relies on more basic modes of sharing, such as familiarity with the emotional and mental states per se (e.g., sadness, embarrassment) that are recognized in basic features of the narrators’ voices.

## 2. Materials and Methods

This study and its hypotheses were preregistered on the Open Science Framework (https://doi.org/10.17605/OSF.IO/2S9DR). It was conducted in accordance with the Declaration of Helsinki, and the protocol was approved by the Ethics Committee of the University of Würzburg (Vote GZEK 2023-22). All subjects gave their informed consent for inclusion before they participated in the study.

### 2.1. Sample

We recruited a total of 63 adult participants, comprising 32 individuals with visual impairment and 31 with unimpaired visual abilities. One of the participants decided to drop out after the initial trials, while in a separate instance, we had to terminate the procedure due to technical difficulties. Five participants were excluded because there were serious doubts about their understanding of the task, either due to statements made during the debriefing (2 participants) or due to notably high error rates following our preregistered criteria of more than two standard deviations above the sample mean (3 additional participants). This left us with a final sample of 56 participants (age range = 20–72, mean age = 46.2, 50% female).

The subsample of 29 visually impaired individuals (age range = 21–70, mean age = 44.7, 52% female) was recruited through multiple channels, including associations for the blind, as well as word-of-mouth referrals. All participants had visual acuity of 0.15 or less, with 14 individuals reporting total blindness. In the case of most individuals within the sample (19 participants), the respective visual impairment had been present since birth. The subsample of 27 visually unimpaired individuals matched the demographic characteristics of the visually impaired subsample (age range = 20–72, mean age = 47.9, 48% female) and was recruited through the university’s online recruiting platform, personal contacts of the researchers, and local internet groups. Before participating, all subjects confirmed that they did not meet any of the exclusion criteria, i.e., that they did not suffer from hearing impairment, had not undergone any neurological or psychiatric treatment in the past two years, were not taking medications that could potentially affect their cognitive abilities, and had not been diagnosed with an intellectual disability. All participants spoke German as their native language. They received financial compensation for their participation.

### 2.2. Study Materials

Our design was based on the EmpaToM task (Kanske et al. 2015), which utilizes short video clips in which actors depict individuals (in the following referred to as narrators) recounting short autobiographic stories from their everyday lives. These narrations (10 to 15 s in length) either deal with neutral (e.g., hobbies, work routines) or negative topics (e.g., sickness, loss; Valence manipulation) and give rise to a question either requiring ToM (asking about mental states that have to be inferred) or factual reasoning (asking about facts that have to be inferred; Question Type manipulation). Each narrator contributes four stories, one for each combination of conditions (Valence × Question Type). After every narration, a rating of the current affect (Affect Rating; ‘How are you feeling?’) and the performance in the ToM/factual reasoning question (Accuracy, Reaction Time) are recorded.

**Figure 1:**
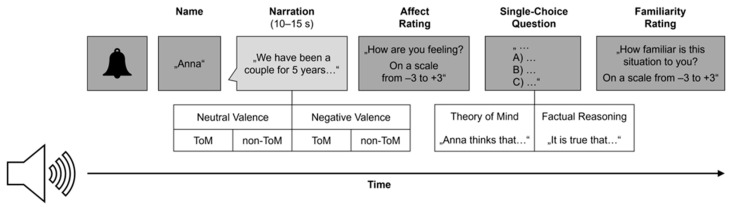
Schematic trial procedure. Trials were exclusively presented in auditory format. Instructions were delivered by the same female voice throughout the trials. Narrations were voiced by one of a total of 20 narrators and had either neutral or negative content (Valence manipulation) and gave rise to a question that required ToM or factual reasoning (Question Type manipulation). Participants gave their responses using a keyboard with marked keys.

The present study deviated from the original procedure in two ways (for a schematic trial procedure, see Figure 1). Firstly, we exclusively used audio tracks without accompanying visual stimuli to ensure equal accessibility of information to all participants, irrespective of their visual impairment. Secondly, we modified and expanded the existing pool of narrations to represent perspectives from both individuals with and without visual impairment (for examples of narrations, see Supplement S1). For narrators without visual impairments, we selected and isolated the audios of existing narrations from the EmpaToM that centered around activities or descriptions only accessible to individuals with unimpaired sight (e.g., driving a car, playing pantomime, describing subtle facial expressions, etc.). In some instances, we combined narrations originally told by different narrators or composed entirely new narrations, which we then had (re-)recorded. Additionally, we generated new material for ten visually impaired narrators (four narrations each, according to the four conditions). These narrations were inspired by firsthand accounts of everyday experiences that visually impaired individuals shared with one of the authors. In addition, individuals with visual impairments provided feedback concerning the plausibility of the narrations. We adapted the accounts to match the existing stories in terms of format and emotional intensity of the topics. Both in narrations by visually unimpaired and impaired narrators, it was possible to identify the narrator’s visual abilities. However, while the visual impairment was the central focus of some narrations (e.g., describing an accident leading to a loss of vision), it played an important but less essential part (e.g., affecting the likelihood or emotional impact of experiences) or an incidental role in others. This deliberate choice was made to construct an ecologically valid range of experiences and avoid reducing visually impaired narrators to their impairment or to mainly passive ‘victims’. Additionally, we ensured that the ToM and factual reasoning questions did not significantly differ from the existing questions concerning key linguistic features (e.g., number of words, past tense, conditional sentences). The narrations were impersonated and recorded by amateur actors. From the total pool of 80 stories by 20 narrators, we curated 6 stimuli sets of 40 stories (10 narrators) each, ensuring an equal ratio of visually impaired and visually unimpaired, as well as male and female narrators. Additionally, we made sure that stories with high thematic similarities (e.g., injuring another person in a car accident) were not presented together in any set. Participants were randomly assigned to one of these sets.

In order to assess the comparability between the original EmpaToM narrations/questions (individuals without visual impairment) and the newly developed narrations/questions (individuals with visual impairments), we conducted an online pilot study (see Supplement S2 for detailed information). We recruited a total of 30 visually unimpaired participants from the platform Prolific (www.prolific.co, accessed on 9 June 2023), with one participant being excluded from data analysis due to their response accuracy falling below chance level (final sample: mean age = 31.2, 55% female). Each participant completed 40 trials of the EmpaToM paradigm, where the narrations were presented as audio clips, while the instructions and questions were presented in written form. A 2 × 2 × 2 repeated-measures Analysis of Variance (ANOVA; *Visual Ability of Narrator* × *Valence* × *Question Type*) revealed no main effect or interaction indicating significant differences between stories by narrators with visual impairment (new narrations) and without visual impairment (original narrations) regarding the affect rating (Main Effect *Visual Ability of Narrator* and Interaction *Valence × Visual Ability of Narrator*: *F* < 1). However, questions linked to stories from visually impaired narrators (new stories) were answered with significantly higher accuracy than those from visually unimpaired narrators (Main Effect *Visual Ability of Narrator*: *F*(1,28) = 58.31, *p* < .001). In response to these findings, we adjusted the difficulty of several questions from the newly or re-recorded narrations.

### 2.3. Procedure

The experiment was programmed and conducted with PsychoPy, version 2022.2.5 (Peirce et al. 2019). To create equal conditions for all participants, instructions and questions were presented exclusively via audio recordings featuring a German-speaking, young, female voice. Laptops (Lenovo, Dell, Medion) were specifically prepared with raised markings on three keys (F, J, and Ö on a German standard keyboard) and over-ear headphones. Data collection took place either in the university’s rooms or at locations more easily accessible to the participants (e.g., their homes). The participants took part in the study individually or, at most, two at a time.

As part of the instructions, participants were made aware that they would be presented with narrations from individuals both with and without visual impairments, under the pretense that these stories were compiled from previous studies. They were asked to concentrate on the content of the narrations while being informed that their task was not to determine whether a specific narrator had visual impairments or not. Subsequently, participants received instructions on how to utilize the marked keyboard to provide their answers. For questions requiring a rating, the marked keys represented the endpoints of a seven-point scale (left: −3, middle: 0, right: 3), while the two unmarked keys in between the marked keys represented the middle points (e.g., the first unmarked key on the left: −2, the second unmarked key on the left: −1, etc.). For multiple-choice questions, the marked keys corresponded to the three answer options (left: A, middle: B, right: C). After practicing the use of the keyboard, participants proceeded to complete two practice trials. If they had no questions, the experimental trials started, presented in randomized order.

Each trial (Figure 1) was initiated by a beep, followed by the narrator’s name. Participants then heard the audio clip once (10–15 s). Afterward, they rated their affect (‘How are you feeling?’) on a scale from −3 (‘very bad’) to +3 (‘very good’) (*Affect Rating*). Next, participants were asked to select the correct statement about the narration’s content out of three options. Identifying the correct option required either factual reasoning (‘It is true, that…’) or ToM (e.g., ‘Anna thinks, that…’). If needed, participants had the opportunity to listen to the options once more by pressing the space bar. We recorded whether participants answered the question correctly (*Accuracy*) as well as their response time (*Reaction Time*) in trials in which they provided the correct answer after hearing the options once. Finally, participants were asked to indicate their level of familiarity with the situation on a scale from −3 (‘not at all familiar’) to +3 (‘very familiar’) (*Familiarity Rating*). After participants had completed all 40 trials, they were asked about their assumptions regarding the purpose of the study. They were then debriefed and thanked for their participation.

### 2.4. Design and Analysis

We performed 2 × 2 × 2 × 2 mixed ANOVAs including the following factors: *Visual Ability of Participant* (visually unimpaired vs. impaired) as between-factor and *Visual Ability of Narrator* (visually unimpaired vs. impaired), *Valence* of the narration (neutral vs. negative), and *Question Type* (ToM vs. factual reasoning) as within-factors. We conducted separate analyses for the three dependent variables, *Affect Rating*, *Accuracy*, and *Reaction Time,* and examined significant interaction effects using additional ANOVAs and *t*-tests. We applied Bonferroni–Holm correction to *t*-tests exploring the same effect to adjust for alpha inflation.

We also conducted a similar four-factorial ANOVA for the variable *Familiarity* to assess whether narrations from the perspective of visually impaired individuals were indeed perceived as more familiar by participants who were visually impaired themselves and vice versa. In addition, we computed correlations between familiarity ratings and affect ratings within negative trials, as well as between familiarity ratings and accuracy overall.

Following the main analyses, we conducted a series of exploratory analyses to examine whether specific characteristics within our sample or our stimulus material significantly influenced or distorted the results. Firstly, we reran the main analyses, excluding the five participants who had correctly guessed the study’s aim in order to eliminate potential biases. Secondly, we explored potential differences among the visually impaired participants in our sample with regard to the duration of their impairment (congenital vs. acquired) and reconducted the main analyses, including only the 19 participants of this subgroup whose impairment was congenital. Additionally, we investigated the potential impact of heterogeneity in our narrations regarding the centrality of visual abilities (for a more detailed explanation, see Supplement S5). To this end, we conducted additional analyses, excluding 26 out of the 80 narrations where the narrator’s visual abilities were incidental to the described events and did neither enable nor substantially influence the experience.

All analyses were conducted using R, version 4.2.2 (R Core Team 2022), in conjunction with the packages rstatix (Kassambara 2022) and afex (Singmann et al. 2023).

## 3. Results

The data presented in this study are openly available on the OSF (https://doi.org/10.17605/OSF.IO/V2WQY).

### 3.1. Affect Rating

**Figure 2:**
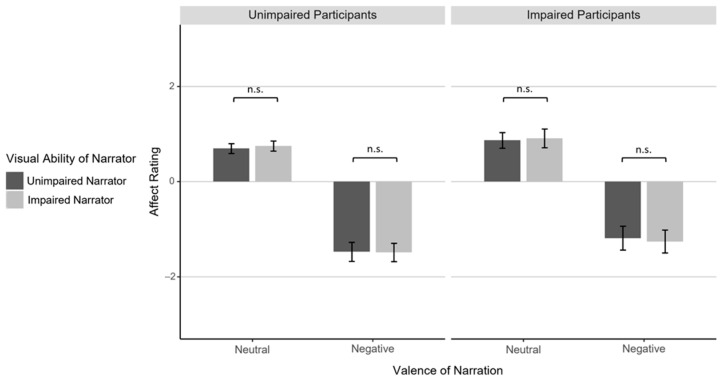
Mean affect ratings given by visually unimpaired and impaired participants (left/right plot) after neutral and negative narrations (left/right two columns in each plot) by visually unimpaired and impaired narrators (dark gray/light gray columns). Error bars indicate standard errors. Horizontal brackets indicate pairwise comparisons: n.s.: *p* >= .05. In addition to the pairwise comparisons highlighted in the figure, affect ratings did not differ between unimpaired and impaired participants for any of the Narrator and Valence conditions (all *p*s > .05).

Confirming the effectiveness of our empathy induction, we found a strong main effect of Valence, *F*(1,54) = 259.89, *p* < .001, η_p_² = .83, with lower affect ratings following negative (*M* = −1.35, *SD* = 1.16) compared to neutral narrations (*M* = 0.81, *SD* = 0.72; see Table 1 for an overview). Critically, and contrary to Hypothesis A, empathic resonance was not enhanced when participants shared the visual experience with the narrators, as reflected in the absence of a three-way interaction between Visual Ability of Participant, Visual Ability of Narrator, and Valence, *F* < 1 (see Figure 2). There were no main effects of Visual Ability of Participant or Visual Ability of Narrator, and no two-way interaction between Visual Ability of Participant and Valence or between Visual Ability of Narrator and Valence, all *F*s < 1. Hence, the visual abilities of the participants and of the narrators did not systematically affect empathic responding.

Some additional effects unrelated to our hypotheses were found: the main effect of Question Type reached significance, *F*(1,54) = 11.68, *p* = .001, η_p_² = .18, with participants giving slightly lower affect ratings in trials involving ToM reasoning (*M* = −0.35, *SD* = 0.89) than in trials requiring factual reasoning (*M* = −0.19, *SD* = 0.80). This is in line with earlier findings (Kanske et al. 2015), as is the interaction between Valence and Question Type, *F*(1,54) = 67.85, *p* < .001, η_p_² = .56. Specifically, previous studies employing the standard EmpaToM have reported that affect ratings in the neutral condition tend to be lower for ToM trials than those for factual reasoning trials, with no significant differences in the negative condition. Our study replicated this pattern for narrations by visually unimpaired narrators, i.e., stories that were adapted from the original EmpaToM with little or no changes, *F*(1,55) = 21.18, *p* < .001, η_p_² = .28. For narrations from visually impaired narrators, i.e., newly generated stories, we observed an interaction, *F*(1,55) = 55.09, *p* < .001, η_p_² = .50, in the form of (in terms of absolute values) stronger affect ratings for factual reasoning trials compared to ToM trials in both valence conditions, i.e., lower ratings in negative trials, *t*(55) = 6.19, *p* < .001, *d* = 0.83, and higher ratings in neutral trials, *t*(55) = 6.55, *p* < .001, *d* = 0.88. This was reflected in a significant three-way interaction of Visual Ability of Narrator × Valence × Question Type, *F*(1,54) = 15.90, *p* < .001, η_p_² = .23, as well as a significant two-way interaction of Visual Ability of Narrator × Question Type, *F*(1,54) = 8.63, *p* = .005, η_p_² = .14. As indicated by a small, but significant four-way interaction, *F*(1,54) = 6.39, *p* = .014, η_p_² = .11, the visually impaired subsample deviated from the described pattern specifically for negative stories told by visually unimpaired narrators by reporting less negative affect after factual reasoning compared to ToM trials, *t*(28) = 2.15, *p* = .040, *d* = 0.40. No other interactions reached significance (all *p*s > .405).

### 3.2. Response Accuracy

Participants answered the questions about the narrations with a mean accuracy of 0.75 (*SD* = 0.10; see Table 1), indicating that performance was not at the ceiling, similar to earlier implementations of the task (Kanske et al. 2015; Tusche et al. 2016). Also in line with earlier findings, the four-factorial ANOVA revealed a significant main effect of Question Type, *F*(1,54) = 7.18, *p* = .010, η_p_² = .12, as ToM questions (*M* = 0.78, *SD* = 0.11) were slightly easier than factual reasoning questions (*M* = 0.73, *SD* = 0.13). This effect was only present for negative narrations by visually unimpaired narrators (‘old’ stories from the original task), *t*(55) = 4.71, *p* < .001, *d* = 0.63, all other *t*s < 1, reflected in significant interactions between Question Type × Visual Ability of Narrator, *F*(1,54) = 6.58, *p* = .013, η_p_² = .11, and Question Type × Valence, *F*(1,54) = 11.99, *p* = .001, η_p_² = .18, as well as a significant three-way interaction, *F*(1,54) = 5.52, *p* = .023, η_p_² = .09.

Concerning our novel manipulations, we observed a small main effect of Visual Ability of Participant, *F*(1,54) = 4.80, *p* = .033, η_p_² = .08, with slightly lower overall accuracy in the visually impaired (*M* = 0.73, *SD* = 0.09) than in the unimpaired group (*M* = 0.78, *SD* = 0.10). In addition, the main effect of Visual Ability of Narrator, *F*(1,54) = 37.73, *p* < .001, η_p_² = .41, indicated higher accuracy for narrations by visually impaired narrators (newly created narrations; *M* = 0.81, *SD* = 0.11) compared to visually unimpaired narrators (old narrations; *M* = 0.70, *SD* = 0.13). This effect was more pronounced for neutral than for negative narrations, resulting in a significant interaction Visual Ability of Narrator × Valence, *F*(1,54) = 7.26, *p* = .009, η_p_² = .12.

**Figure 3:**
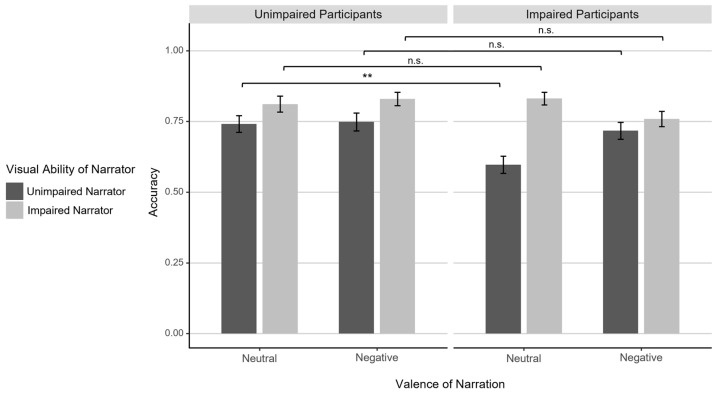
Mean response accuracy for visually unimpaired and impaired participants (left/right plot) for questions about neutral and negative narrations (left/right two columns in each plot) by visually unimpaired and impaired narrators (dark gray/light gray columns). Error bars indicate standard errors. Horizontal brackets indicate pairwise comparisons: n.s.: *p* >= .05, **: *p* < .01.

Critically and contrary to expectations of better mentalizing/reasoning performance when the visual abilities of participant and narrator matched (Hypothesis A), the factors Visual Ability of Participant and Visual Ability of Narrator did not interact, *F*(1,54) = 3.17, *p* = .081, η_p_² = .06. There was, however, a significant three-way interaction between Visual Ability of Participant, Visual Ability of Narrator, and Valence, *F*(1,54) = 9.14, *p* = .004, η_p_² = .14, pointing toward the predicted effect when participants with visual impairments listened to neutral narrations of unimpaired speakers (see Figure 3). Post hoc *t*-tests comparing the visually impaired and unimpaired subsamples indicated that visually impaired participants displayed notably lower accuracy for neutral narrations by unimpaired individuals, *t*(54.0) = 3.41, *p* = .005, *d* = 0.91, while performance did not significantly differ between the groups for negative narrations or narrations by visually impaired individuals, *p*s > .160. No other main effects or interactions reached significance, *p*s > .200.

### 3.3. Reaction Time

Reaction times were analyzed for trials with correct responses in which participants had listened to the answer options only once. We observed a main effect of Visual Ability of Participant, *F*(1,53) = 5.78, *p* = .020, η_p_² = .10, with slower reaction times for visually impaired (*M* = 2.14, *SD* = 0.95) compared to visually unimpaired participants (*M* = 1.65, *SD* = 0.47) (for an overview see Table 2). Additionally, answering questions about narrations from visually unimpaired narrators took longer (*M* = 2.03, *SD* = 0.95) compared to narrations by visually impaired narrators (*M* = 1.76, *SD* = 0.75), *F*(1,53) = 7.86, *p* = .007, η_p_² = .13. Hence, effects on reaction times were consistent with accuracy findings, rendering a speed–accuracy tradeoff unlikely. No other main effects or interactions reached significance (all *p*s > .166).

### 3.4. Familiarity Rating

In order to conduct manipulation checks and exploratory analyses, participants rated their familiarity with the recounted situation in every trial. The four-factorial ANOVA on these ratings showed a significant main effect of Valence, *F*(1,54) = 78.62, *p* < .001, η_p_² = .59, as all participants considered neutral narrations as more familiar (*M* = 0.02, *SD* = 1.12) than negative ones (*M* = −0.99, *SD* = 1.19; see Table 2). The significant main effect of Question Type, *F*(1,54) = 7.08, *p* = .010, η_p_² = .12, was due to higher familiarity ratings for narrations in the ToM condition (*M* = −0.37, *SD* = 1.12) compared to the factual reasoning condition (*M* = −0.60, *SD* = 1.10). This latter effect was only significant for negative narrations by visually impaired narrators, *t*(55) = 3.48, *p* = .004, *d* = 0.46, all other *p*s > .435, resulting in a significant three-way interaction of Valence × Visual Ability of Narrator × Question Type, *F*(1,54) = 5.92, *p* = .018, η_p_² = .10.

**Figure 4:**
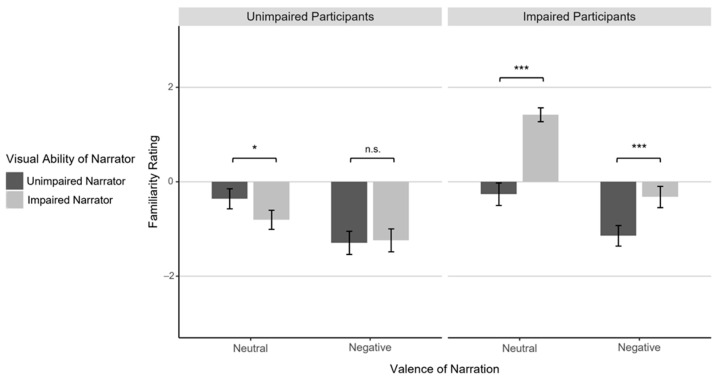
Mean familiarity ratings given by visually unimpaired and impaired participants (left/right plot) after neutral and negative narrations (left/right two columns in each plot) by visually unimpaired and impaired narrators (dark gray/light gray columns). Error bars indicate standard errors. Horizontal brackets indicate pairwise comparisons: n.s.: *p* >= .05, *: *p* < .05, ***: *p* < .001.

Concerning our main manipulations, participants with visual impairment gave overall higher familiarity ratings, *F*(1,54) = 10.36, *p* = .002, η_p_² = .16 (*M* = −0.08, *SD* = 0.96 vs. *M* = −0.92, *SD* = 1.01), and narrations by visually impaired narrators were rated more familiar, *F*(1,54) = 34.37, *p* < .001, η_p_² = .39 (*M* = −0.21, *SD* = 1.26 vs. *M* = −0.76, *SD* = 1.09). As predicted, these factors interacted, *F*(1,54) = 64.31, *p* < .001, η_p_² = .54. Visually impaired participants gave markedly higher familiarity ratings for stories by narrators who were themselves visually impaired compared to those who were not, *F*(1,28) = 92.65, *p* < .001, η_p_² = .77. Visually unimpaired participants showed an according matching advantage (higher familiarity with narrations from unimpaired speakers), but only in the neutral condition, *t*(26) = 2.73, *p* < .022, *d* = 0.53, and not the negative one, *t* < 1. This weaker matching effect in visually unimpaired participants was reflected in a significant three-way interaction of Visual Ability of Participant × Visual Ability of Narrator × Valence, *F*(1,54) = 23.20, *p* < .001, η_p_² = .30 (see Figure 4), as well as a significant two-way interaction of Visual Ability of Participant × Valence, *F*(1,54) = 7.78, *p* = .007, η_p_² = .13. No further interactions reached significance (*p*s > .103).

For exploratory purposes, we conducted correlation analyses on the trial-by-trial data to examine connections between familiarity with a specific situation and our main dependent variables. We found that higher familiarity ratings were neither associated with lower affect ratings after negative trials, *r*(1118) = −.03, *p* = .283, nor with higher accuracy, *r*(2238) = .01, *p* = .618. Overall, participants did not display higher levels of empathy or reasoning proficiency when they were familiar with a given situation. Because familiarity ratings differed markedly between visually impaired and unimpaired participants, we additionally performed separate analyses for the two participant groups. Among visually unimpaired participants, higher familiarity ratings in negative trials were linked to slightly lower affect ratings, *r*(538) = −.21, *p* < .001, suggesting a heightened emotional response to familiar situations. We observed no comparable effect for the visually impaired subsample, *r*(578) = .06, *p* = .121. In contrast, higher familiarity ratings showed a small positive correlation with response accuracy in visually impaired, *r*(1158) = .06, *p* = .046, but not unimpaired participants, *r*(1078) = −.02, *p* = .489. The latter effect, though notably small, seems in line with the reduced accuracy that visually impaired participants achieved for neutral narrations by visually unimpaired speakers.

### 3.5. Exploratory Analyses

In the following, we will report the results of three additional analyses: (a) excluding participants who guessed the goal of the study, (b) including only congenitally blind participants, and (c) including only narrations in which visual impairment was substantial or essential to the narration. We will focus on the effects relevant to our hypotheses, i.e., a potential advantage for social understanding if visual abilities match between participant and narrator. Concerning empathy, i.e., Affect Rating as dependent variable, this effect should manifest in a significant interaction of Visual Ability of Participant × Visual Ability of Narrator × Valence. Concerning Theory of Mind, i.e., Response Accuracy, it should be reflected in a significant interaction of Visual Ability of Participant × Visual Ability of Narrator. For Response Accuracy, we will also provide details on the interaction of Visual Ability of Participant × Visual Ability of Narrator x Valance, where significant differences were noted in the main analysis. A full overview of the results is available in the Supplements (Supplements S3–S5).

We reconducted the main analyses after excluding the five participants who correctly guessed the study’s aim. The overall, non-significant pattern of results regarding a potential matching effect persisted for both Affect Rating, *F*(1,49) < 1, and for Response Accuracy, *F*(1,49) = 2.12, *p* = .152, $\eta_{p}^{2}$ = .04, while the three-way interaction of Visual Ability of Participant × Visual Ability of Narrator × Valence for Response Accuracy remained statistically significant, *F*(1,49) = 7.72, *p* = .008, $\eta_{p}^{2}$ = .14. The same was true for a second exploratory analysis, which included only the 19 participants whose impairment was congenital (vs. acquired) in the visually impaired subgroup: we found no interactions indicating an overall matching effect (Affect Rating: *F*(1,44) < 1; Response Accuracy: *F*(1,44) = 2.36, *p* = .132, $\eta_{p}^{2}$ = .05), but a significant interaction of Visual Ability of Participant × Visual Ability of Narrator × Valence for Response Accuracy, *F*(1,44) = 12.35, *p* = .001, $\eta_{p}^{2}$ = .22.

Furthermore, we repeated the main analyses, excluding 26 narrations in which the narrator’s visual abilities were incidental to the described events. Compared to the analyses including the complete stimulus set, the pattern relevant to our hypothesis remained unchanged, meaning that we observed no significant overall matching advantage for Affect Ratings, *F*(1,54) < 1, or Response Accuracy, *F*(1,54) = 2.82, *p* = .099, $\eta_{p}^{2}$ < .05. For Response Accuracy, the three-way interaction of Visual Ability of Participant × Visual Ability of Narrator × Valence was again highly significant, *F*(1,54) = 17.26, *p* < .001, $\eta_{p}^{2}$ < .24. This consistency also persisted in additional analyses focusing specifically on a subset of narrators, in whose narrations the centrality of visual abilities was particularly pronounced (see Supplement S5).

## 4. Discussion

Understanding another person’s perspective and emotions involves complex and intricate processes, yet humans often master this challenge successfully. What underpins this remarkable capacity for empathizing and mentalizing? Some would argue that individuals ground their understanding of another person in their own perceptual and life experiences (e.g., Barsalou 2008; Dimaggio et al. 2008; Gallese and Goldman 1998; Meltzoff 2007). But to what degree do experiences need to be familiar? Our study compared two groups of participants differing in a central feature, namely, their visual ability, in terms of their empathy and cognitive perspective-taking with narrators who were themselves visually impaired or unimpaired. Overall, our results did not reveal consistent benefits in empathic responses and ToM performance when the narrator’s visual abilities (and, therefore, presumably, their life experiences) aligned with those of the participant. Hence, firsthand familiarity with a specific situation is not a necessary precondition for social understanding. To elaborate on this overall conclusion, it is worth taking a more differentiated look at the two core components of social understanding, empathy and ToM, and how they were shaped by the visual ability manipulations in the present study.

### 4.1. Empathy

Replicating earlier implementations of the EmpaToM, participants reported more negative affect after negative than after neutral narrations (Kanske et al. 2015; Tholen et al. 2020; Tusche et al. 2016). This finding demonstrates that the novel and auditory-only adaptation of the task can successfully induce empathy, i.e., the sharing of another’s affect (De Vignemont and Singer 2006). Critically, empathic responding in the present study did not depend on the (mis-)match of visual abilities between participant and narrator (supporting Hypothesis B). All participants exhibited robust and comparable empathic reactions irrespective of their own and the narrator’s visual abilities, i.e., regardless of whether the scenario could potentially occur in their own reality of life. Therefore, affective responses did not depend on firsthand experience with the described situation (Danziger et al. 2009; Lamm et al. 2010).

On what grounds was empathy elicited in participants, if not through recollection of a similar (painful) experience? Taking into account humans’ ability to recognize emotions from a variety of cues, such as facial expressions, body posture, gaze, or (as in our case) vocal information (Dael et al. 2012; Ekman and Friesen 1971; Scherer 1986), it seems likely that participants discerned immediate emotional cues in the narrations, whether these signals were obvious, like sobbing, or more subtle variations in tone of voice and speech pattern. Historically, voice processing has been studied in much less depth and detail than face processing (Schirmer and Adolphs 2017). However, especially for highly changeable social information such as emotions, voice cues can be crucial (Young et al. 2020), potentially even surpassing visual cues in their significance for empathic accuracy (Kraus 2017). In our study, emotional signals conveyed through voice cues were powerful enough to overshadow the relevance of the participants’ specific experiences and the narrator’s personal characteristics and group belongingness for empathically simulating the narrator’s emotional state. Using realistic and rich auditory stimuli might have also contributed to the divergence of our results from studies reporting a ‘similarity benefit’ in empathy, many of which used written information (e.g, Heinke and Louis 2009; Nelson et al. 2003; Tarrant et al. 2009).

### 4.2. Cognitive Understanding

In our auditory-only adaptation of the EmpaToM, participants’ response accuracy for ToM and factual reasoning was 78% and 73%, respectively, suggesting that similar to past studies employing the original EmpaToM (Kanske et al. 2015; Trautwein et al. 2020; Tusche et al. 2016) the interpretability of our results was not compromised by ceiling effects. Overall, we did not observe general advantages for ToM (as the cognitive component of social understanding; Frith and Frith 2005, 2006) or factual reasoning when the narrator’s visual abilities aligned with those of the participant (supporting Hypothesis B). Hence, even though their realities of life differ in various and meaningful ways, people with and without visual impairments managed, on average, to understand each other’s narrations and correctly deduce related facts and mental states.

However, in specific conditions, a mismatch in life experiences seems to have been detrimental to successfully understanding the other person’s narration. Our subsample of visually impaired participants encountered more difficulties in accurately comprehending neutral stories when the narrator did not share their visual impairment. This effect was observed for both ToM and factual reasoning, and it is therefore unlikely that it stemmed from a specific challenge related to mentalizing. Instead, the lack of experience regarding specific situations may have impeded an exact understanding of the circumstances. This could also provide an explanation for why this effect emerged exclusively for neutral narrations: while negative narrations more often centered on common human experiences such as loss, rejection, failure, or illness, neutral narrations revolved around specific jobs, ventures, daily routines, or hobbies, potentially requiring more in-depth knowledge about the topics. Why, then, did we not observe a similar effect in visually unimpaired participants when they listened to narrations by visually impaired speakers in the present study? One possibility is that the overall lower difficulty of the trials involving visually impaired narrators might have prevented a corresponding effect for visually unimpaired participants. Taken together, like empathy, cognitive perspective-taking appears to be possible even when an interaction partner recounts situations that one has not personally experienced (e.g., Van Boven and Loewenstein 2005). However, unlike empathy, understanding the details and mental states involved in another’s experience may, in some circumstances, benefit from recognizing and remembering a similar situation oneself (Buckner and Carroll 2007; Dimaggio et al. 2008; Gerace et al. 2015).

### 4.3. Perceived Familiarity of the Narrations

Finally, participants rated how familiar they were with the recounted situation in every given trial. While visually impaired participants perceived narrations by visually impaired individuals on average as more familiar than those by narrators without visual impairment, visually unimpaired participants showed less consistent differences in familiarity ratings between visually impaired and unimpaired narrators. Since participants were not provided with an explicit definition of ‘familiar’ in this context, their criteria might have systematically differed. It is reasonable to expect that visually impaired individuals may have a heightened awareness of their group identity, given their membership in a minority group (Brewer 1991; Brewer and Weber 1994; Sekaquaptewa et al. 2007; Smith and Leach 2004). Consequently, they might have assessed similarity on the group level, perceiving narrators who belonged to their group as more closely aligned to their own reality of life. In contrast, visually unimpaired participants likely did not consciously identify themselves as part of a specific group during participation and therefore might have focused on the level of concrete situations for their evaluation of familiarity. Given that even within the EmpaToM narrations by sighted individuals, there is a wide range of demographic characteristics and life circumstances, visually unimpaired participants might have considered these to be just as dissimilar to their own specific life as narrations by visually impaired narrators.

Overall, the subjective degree of familiarity with a situation showed only small correlations, if any, with empathic resonance and mentalizing/factual reasoning accuracy: visually unimpaired participants were slightly more affected by negative incidents they were personally familiar with, while visually impaired participants showed a tendency toward better reasoning in situations they knew themselves. Together with the previously outlined effects of shared visual abilities on social understanding, these findings demonstrate that people, overall, have a stable tendency to empathize and mentalize with others even when they are notably different from themselves. However, shared personal experience can benefit social understanding in some situations.

### 4.4. Limitations

The present study did not control or measure how much attention participants paid to the visual abilities of the narrator as they listened to the narration. It is possible, for instance, that some participants categorized narrators based on their visual (dis-)ability in every trial. Even though we instructed participants to focus on the content of the narrations instead of the narrators’ visual ability, a rating conducted in our pilot study indicated that approximately 89% of participants thought, at least to some extent, about whether narrators were visually impaired or not. Some participants, hence, may have approached the narrations with an element of preoccupation or preconception.

An additional limitation of the present study is the inherent impossibility of encapsulating something as complex and extensive as a person’s reality of life within a few brief narrations. Our narrations only covered a small portion of what constitutes and distinguishes the everyday experiences of visually impaired and unimpaired individuals. Additionally, people’s lives diverge in countless ways, also among individuals sharing significant similarities like visual impairment. Even though our newly generated narrations were based on real-life experiences of visually impaired individuals, they were finalized by a team of researchers without visual impairments, and they cannot represent the reality and experiences of every person falling within that broad category. Another noteworthy aspect concerning our narrations is the inclusion of narrations where the narrator’s visual abilities only played an incidental part. This decision was meant to reduce repetition and fatigue in participants as well as to implement comparable and realistic ranges of experiences in narrators with and without visual impairments. Of course, this could potentially diminish the effects of group differences, given that the essence of the narration revolved around experiences shared by both groups. However, exploratory analyses suggested that, in our study, the match of visual abilities between participant and narrator did not significantly affect empathic responses or cognitive understanding, even when the visual abilities or impairments were central to the described events. Since this observation in our study was post hoc, future research should systematically manipulate this aspect to draw more reliable conclusions.

Furthermore, our study’s generalizability and interpretability are constrained by the modest sample size of only 30 participants per group as well as the diversity within the visually impaired participant group, encompassing both individuals with congenital and acquired visual impairments. It is worth considering that the duration of the impairment might affect both an individual’s life experiences and performance across various tasks. Previous evidence suggests differences between people with congenital and acquired visual impairment, e.g., regarding neurological and perceptual processes as well as mental health (e.g., Büchel et al. 1998; Choi et al. 2019; Monegato et al. 2007; Qin et al. 2015), though there are no conclusive results regarding aspects of social cognition (e.g., Adenzato et al. 2006; Ardito et al. 2004). The inclusion of both groups in our study might carry the risk of conflating differential effects for these subgroups, although exploratory analyses did not yield clear empirical evidence supporting this concern. Given that congenitally impaired individuals constituted two-thirds of our participants and the results remained consistent when excluding participants with acquired impairments, it is conceivable that our findings were predominantly influenced by this group. Consequently, our interpretation may primarily apply to individuals with congenital impairments. Due to the small number of participants with acquired visual impairment in our study, we are cautious to make conclusions about their similarities or differences compared to congenitally impaired participants in the examined processes. Together with the previously discussed limitations, this clearly points out the need for further research including larger samples and more diverse measures of social understanding.

## 5. Conclusions

Despite these limitations, we believe that our study allows some interesting initial conclusions. Firstly, empathy remained unaffected by whether or not visual abilities were shared between participants and narrators, suggesting that affective responses did not rely on perceptual familiarity with specific situations but were grounded in the recognition and simulation of emotional states. Through emotional signals in our auditory stimuli, participants might have been able to efficiently recognize the narrator’s emotional state and to empathize based on their familiarity with the emotion per se, transcending the specific context. Second, while ToM performance and factual reasoning were not generally shaped by the match or mismatch between individuals’ realities of life, visually impaired participants encountered greater difficulty in discerning facts and mental states from neutral narrations when they stemmed from a person with a divergent reality of life. This observation indicates that in certain cases, cognitive inferences and perspective-taking are facilitated by or grounded in personal experience with comparable situations. Hence, in line with a distinction between empathy and ToM that has been suggested by both neurological and behavioral studies (Kanske et al. 2015; McDonald et al. 2022; Trautwein et al. 2020), the effect of shared visual abilities differed between empathy and cognitive understanding. Overall, however, our findings underscore the notion that individuals have the capacity to compensate for discrepancies in perceptual experiences and specific circumstances and derive their social understanding from more basic, fundamental shared experiences and emotions. In the grand scheme of things, being familiar with the relevant psychological states seems to hold more importance for empathizing with and understanding others than having experienced the same event or incident oneself. So even though our neighbor might walk the streets differently than we do, this does not seem to impede our understanding when they tell us about their notion of leaving the house on a crisp autumn morning.

## Figures and Tables

**Table 1:** Means and standard deviations of Affect Rating (left) and Response Accuracy (right) by Visual Ability of Participant, Visual Ability of Narrator, Valence of Narration, and Question Type.

	Affect Rating: Mean (SD)			Accuracy: Mean (SD)		
	Overall	Unimpaired Participants	Impaired Participants	Overall	Unimpaired Participants	Impaired Participants
Overall	−0.27 (0.83)	−0.38 (0.67)	−0.17 (0.96)	0.75 (0.10)	0.78 (0.10)	0.73 (0.09)
Unimpaired Narrators	−0.27 (0.84)	−0.39 (0.68)	−0.16 (0.96)	0.70 (0.13)	0.74 (0.13)	0.66 (0.12)
Neutral Valence	0.79 (0.74)	0.70 (0.54)	0.87 (0.88)	0.67 (0.17)	0.74 (0.15)	0.60 (0.16)
ToM	0.53 (0.88)	0.38 (0.75)	0.67 (0.98)	0.66 (0.22)	0.73 (0.22)	0.59 (0.20)
FR	1.04 (0.79)	1.01 (0.60)	1.07 (0.94)	0.67 (0.2)	0.75 (0.16)	0.60 (0.21)
Negative Valence	−1.32 (1.20)	−1.47 (1.03)	−1.19 (1.34)	0.73 (0.16)	0.75 (0.16)	0.72 (0.16)
ToM	−1.34 (1.23)	−1.41 (1.08)	−1.28 (1.36)	0.82 (0.18)	0.83 (0.17)	0.82 (0.20)
FR	−1.31 (1.22)	−1.54 (1.02)	−1.10 (1.36)	0.64 (0.25)	0.67 (0.25)	0.61 (0.24)
Impaired Narrators	−0.27 (0.86)	−0.37 (0.67)	−0.17 (1.01)	0.81 (0.11)	0.82 (0.11)	0.80 (0.11)
Neutral Valence	0.83 (0.84)	0.75 (0.54)	0.91 (1.06)	0.82 (0.13)	0.81 (0.15)	0.83 (0.12)
ToM	0.51 (0.96)	0.50 (0.66)	0.52 (1.19)	0.81 (0.16)	0.82 (0.17)	0.81 (0.15)
FR	1.16 (0.88)	1.00 (0.61)	1.30 (1.06)	0.83 (0.19)	0.80 (0.20)	0.86 (0.18)
Negative Valence	−1.37 (1.15)	−1.49 (1.00)	−1.26 (1.29)	0.79 (0.14)	0.83 (0.12)	0.76 (0.14)
ToM	−1.10 (1.15)	−1.23 (1.01)	−0.98 (1.27)	0.80 (0.18)	0.85 (0.15)	0.76 (0.20)
FR	−1.64 (1.25)	−1.75 (1.05)	−1.54 (1.42)	0.78 (0.18)	0.81 (0.16)	0.76 (0.19)

ToM = Theory of Mind; FR = Factual Reasoning.

**Table 2:** Means and standard deviations of Reaction Time in s (left) and Familiarity Rating (right) by Visual Ability of Participant, Visual Ability of Narrator, Valence of Narration, and Question Type.

	Reaction Time: Mean (SD)			Familiarity Rating: Mean (SD)		
	Overall	Unimpaired Participants	Impaired Participants	Overall	Unimpaired Participants	Impaired Participants
Overall	1.90 (0.79)	1.65 (0.47)	2.14 (0.95)	−0.48 (1.06)	−0.92 (1.01)	−0.08 (0.96)
Unimpaired Narrators	2.03 (0.95)	1.72 (0.63)	2.34 (1.11)	−0.76 (1.09)	−0.83 (1.03)	−0.70 (1.15)
*Neutral Valence*	2.02 (1.02)	1.65 (0.63)	2.37 (1.19)	−0.31 (1.19)	−0.36 (1.11)	−0.26 (1.28)
ToM	2.06 (1.67)	1.67 (0.99)	2.42 (2.07)	−0.19 (1.23)	−0.24 (1.11)	−0.14 (1.35)
FR	1.98 (1.14)	1.62 (0.76)	2.32 (1.33)	−0.43 (1.43)	−0.48 (1.41)	−0.38 (1.47)
*Negative Valence*	2.06 (1.22)	1.79 (0.87)	2.32 (1.44)	−1.21 (1.21)	−1.29 (1.27)	−1.14 (1.17)
ToM	1.97 (1.37)	1.68 (1.25)	2.24 (1.44)	−1.21 (1.38)	−1.31 (1.38)	−1.12 (1.41)
FR	2.12 (1.77)	1.90 (1.28)	2.34 (2.13)	−1.21 (1.30)	−1.27 (1.33)	−1.16 (1.29)
Impaired Narrators	1.76 (0.75)	1.58 (0.46)	1.93 (0.93)	−0.21 (1.26)	−1.02 (1.09)	0.55 (0.87)
*Neutral Valence*	1.85 (0.84)	1.67 (0.52)	2.03 (1.03)	0.35 (1.45)	−0.80 (1.05)	1.42 (0.78)
ToM	1.83 (1.24)	1.66 (0.74)	1.99 (1.57)	0.39 (1.46)	−0.70 (1.14)	1.41 (0.87)
FR	1.88 (1.09)	1.67 (0.84)	2.07 (1.27)	0.30 (1.62)	−0.90 (1.25)	1.43 (0.98)
*Negative Valence*	1.67 (0.91)	1.51 (0.60)	1.82 (1.11)	−0.76 (1.31)	−1.24 (1.26)	−0.32 (1.21)
ToM	1.56 (1.20)	1.40 (0.46)	1.71 (1.6)	−0.48 (1.55)	−1.16 (1.40)	0.15 (1.44)
FR	1.77 (1.22)	1.61 (0.97)	1.93 (1.42)	−1.04 (1.32)	−1.31 (1.37)	−0.79 (1.24)

ToM = Theory of Mind; FR = Factual Reasoning.

## Data Availability

The data presented in this study are openly available on the OSF (https://doi.org/10.17605/OSF.IO/V2WQY).

## Supplementary Materials

The following supporting information can be downloaded at: https://www.mdpi.com/article/10.3390/jintelligence12010002/s1. S1: Examples of narrations by visually unimpaired and impaired narrators; S2: Results from the online pilot study; S3: Main analyses, excluding participants who correctly guessed the study aim; S4: Main analyses, including only participants with congenital/acquired impairment in the visually impaired subsample; S5: Exploratory analyses regarding the importance of visual abilities in the narrations.
