# Supplementary material for: Social Understanding beyond the Familiar: Disparity in Visual Abilities Does Not Impede Empathy and Theory of Mind
Source: J Intell. 2023 Dec 25;12(1):2. doi: 10.3390/jintelligence12010002 (PMC10816830; doi:10.3390/jintelligence12010002)
Supplement: Supplementary file 1 [file jintelligence-12-00002-s001.zip › jintelligence-2665752-supplementary.pdf]

Supplementary Materials for

Social understanding beyond the familiar: Disparity in visual abilities does not impede empathy and Theory of Mind

**S1**	Examples of narrations by visually unimpaired and impaired narrators	
**S2**	Results from the online pilot study	
	**Table S2.1**	Analysis of affect rating (pilot study)
	**Figure S2.1**	Mean affect rating (pilot study)
	**Table S2.2**	Analysis of accuracy (pilot study)
	**Figure S2.2**	Mean accuracy (pilot study)
**S3**	Main analyses excluding participants who correctly guessed the study aim	
	**Table S3.1**	Analysis of affect rating excluding participants who correctly guessed the study aim
	**Figure S3.1**	Mean affect rating excluding participants who correctly guessed the study aim
	**Table S3.2**	Analysis of accuracy excluding participants who correctly guessed the study aim
	**Figure S3.2**	Mean accuracy excluding participants who correctly guessed the study aim
**S4**	Main analyses including only participants with congenital/acquired impairment in the visually impaired subsample	
	**Table S4.1**	Analysis of affect rating including only participants with congenital and acquired impairment in the visually impaired subsample
	**Figure S4.1**	Mean affect rating including only participants with congenital and acquired impairment in the visually impaired subsample
	**Table S4.2**	Analysis of accuracy including only participants with congenital and acquired impairment in the visually impaired subsample
	**Figure S4.2**	Mean accuracy including only participants with congenital and acquired impairment in the visually impaired subsample
**S5**	Exploratory analyses regarding the importance of visual abilities in the narrations	
	**Table S5.1**	Analysis of affect rating including only a subset of narrations with visual abilities at their core and a subset of narrators with visual abilities at their core
	**Figure S5.1**	Mean affect rating including only a subset of narrations with visual abilities at their core and a subset of narrators with visual abilities at their core
	**Table S5.2**	Analysis of accuracy including only a subset of narrations with visual abilities at their core and a subset of narrators with visual abilities at their core
	**Figure S5.2**	Mean accuracy including only a subset of narrations with visual abilities at their core and a subset of narrators with visual abilities at their core

S1 Examples of narrations by visually unimpaired and impaired narrators

In total, we used a pool of 80 narrations by 20 narrators (10 visually unimpaired, 10 visually impaired). The narrations differed in their emotional valence (neutral vs. negative) and in the type of question they gave rise to (factual reasoning vs. ToM). The following four exemplary narrations (2 by visually impaired, 2 by visually unimpaired narrators) were transcribed and translated to English from their original German version by the authors. Underlined options indicate the correct answer to the respective question.

Ben (30 years old) – Visually unimpaired

Narration 1 - Neutral & Factual Reasoning
"I was trained as a fitness instructor, and I've been working in a gym for years. I take care of the training plans, but also the nutrition plans and make sure that the customers do their exercises correctly. Well, for the really tough ones I also offer a boot camp from time to time."

It is true,
- <u>that Ben works in a gym that also offers diet counseling.</u>
- that Ben did his training in a studio and has continued to work there.
- that Ben is mainly busy with classes.

Narration 2 - Neutral & ToM
"My girlfriend is training to be a hairdresser. And last week she came to me and said, " Hey, Ben. We learned about a new haircut- I'm sure it would look good on you." But honestly, when I look at her brother..."

Ben thinks,
- <u>that his girlfriend is not yet suitable as a hairdresser.</u>
- that his girlfriend should cut his hair for practice purposes.
- that his girlfriend wants to cut his hair to make him happy.

Narration 3 - Negative & Factual Reasoning
"Yeah, my girlfriend and I are planning to move in together now.... It would be cheaper and closer to the gym where I work... But, she is allergic to animal hair and that's why I have to give away my cat Felix... After 15 years that would be really hard..."

It is true,
- <u>that there are practical reasons for Ben and his girlfriend to move in together.</u>
- that there would be too many animals if Ben didn't give away his cat.
- that the landlord of the new apartment does not allow pets

Narration 4 - Negative & ToM
" We have been trying for three years now and then .... the doctor's report came... I was totally shocked when I read the word ‘infertile’.... and all because of the failed surgery of my hernia... Maybe it is just not meant to be. My girlfriend, on the other hand, almost seemed relieved somehow..."

Ben thinks,
- <u>that Ben's girlfriend doesn't really want to have children.</u>
- that Ben's girlfriend now wants to adopt a child.
- that Ben's girlfriend will now look for a new partner.

Helga (48 years old) – Visually unimpaired

Narration 1 - Neutral & Factual Reasoning
"I was 25 when I finished my apprenticeship as a dressmaker... Then I was employed as an alteration tailor for almost ten years... But since a few years I have my own small boutique. Now I design and sew especially evening wear."

It is true
- <u>that Helga's current job requires more creativity than her previous job.</u>
- that Helga trained as a dressmaker immediately after she finished school.
- that Helga no longer has anything to do with sketching patterns.

Narration 2 - Neutral & ToM
"I have a new intern now, a young guy named Conny. I've never seen anyone develop a knack for fabrics and sewing machines so quickly! And now he thinks his parents want him to study fashion design instead of doing an apprenticeship..."

Helga thinks,
- <u>that an apprenticeship is the right thing for Conny, because he is practically very talented.</u>
- that Conny will not succeed in his studies because he will not be disciplined enough.
- that Conny's parents do not take their son's interests into consideration.

Narration 3 - Negative & Factual Reasoning
"I was on my way home and I was driving along this 20-mph street, like every day... and suddenly a child ran out into the street between the cars. (swallows) I tried to brake... but it all happened way too fast."

It is true,
- <u>that Helga travels by car on weekdays.</u>
- that Helga was driving faster than the speed limit that day.
- that the accident would not have happened if the cars had parked properly.

Narration 4 - Negative & ToM
"I keep picturing the accident...that child under my car...Since then, my life feels so, so wrong, so unjust...whenever I see my daughter...but without her, I would have given up long ago..."

Helga thinks,
- <u>that she suffers so much due to the accident, because she also has a daughter.</u>
- that she needs to seek professional help so that she can properly process the accident.
- that her daughter reminds her in appearance of the child who died.

Anton (34 years old) – Visually impaired

Narration 1 - Neutral & Factual Reasoning
" The other day I went for a walk with Charlie, my guide dog, in the park behind the train station. I could really smell the lilies of the valley and hear the birds chirping. I often go for walks in spring or fall - that's when the smells are just the most intense."

It is true,

- <u>that the walk took place on a spring day.</u>
- that the park is located far from the city center and difficult to reach.
- that Anton was walking a friend's dog.

Narration 2 - Neutral & ToM
"At the park the other day, a woman asked me about my guide dog. She didn't stop talking about how cute he was... and then she snorted at her husband: (mimicking) 'you and your stupid pet hair allergy'."

Anton thinks,

- <u>that the topic of pets is a sore spot in their relationship.</u>
- that the wife thinks her husband should be more considerate of her wishes.
- that the wife thinks her husband is exaggerating the severity of his allergy.

Narration 3 - Negative & Factual Reasoning
"And then the vet put my guide dog down.... He said with so many metastases, there was no point in starting chemo anymore. I stayed with Charlie until the end.... It is unimaginable that he's just not there anymore."

It is true,

- <u>that Anton's dog did not suffer in his final moments.</u>
- that Anton's guide dog died as a result of a severe bout of cancer.
- that the chemotherapy prescribed by the veterinarian did not work for Anton's guide dog.

Narration 4 - Negative & ToM
"Ever since my guide dog Charlie died, my wife has talked nonstop about how we didn't keep any of his stuff at all. No toys that smell like him .... Just nothing to remember him by."

Anton thinks,

- <u>that his wife believes mementos can help with grieving.</u>
- that his wife is afraid Anton isn't thinking enough about Charlie.
- that his wife wants a new pet as soon as possible to replace Charlie.

Marita (48 years old) – Visually impaired

Narration 1 - Neutral & Factual Reasoning
"When I first met my husband's family, I also went to his house for the first time.... I went over to his place a few hours before the family so that I could get oriented, and my husband could at least show me where the bathroom was located..."

It is true,
- <u>that Marita and her husband did not live together when she met his family.</u>
- that Marita had a long-distance relationship before she married her husband.
- that Marita's husband's house is built in such a way that it is hard to find the toilet.

Narration 2 - Neutral & ToM
"When I go to the market, my vegetable vendor always shouts from far away that he'll be right there and that no one else should serve me. (Sighs) I don't really want special treatment because of my visual impairment, but I just do him the favor."

Marita thinks,
- <u>that the vegetable vendor is happy to help her because of her handicap.</u>
- that the vegetable vendor has romantic feelings for her and therefore wants to serve her personally.
- that the vegetable vendor wants to make more sales by giving her special treatment.

Narration 3 - Negative & Factual Reasoning
"My husband once just left me in a foreign city in the winter after one of our many fights ... it took me hours to find my way home... and I was so cold... and I felt so helpless..."

It is true,
- <u>that Marita and her husband argued when they explored a new city.</u>
- that Marita and her husband argued regularly as soon as they were traveling together.
- that Marita and her husband argue every day.

Narration 4 - Negative & ToM
"I lost my eyesight in a car accident... (sobbing) The time after that was (voice breaks) absolute hell... and then the person who caused the accident contacted me and whined to ME, (crying) how sorry HE was!"

Marita thinks,
- <u>that the person who caused the accident contacted her to ease his conscience.</u>
- that the person who caused the accident contacted her because he was honestly interested in her recovery.
- that the person who caused the accident contacted her because he wanted to offer her support.

S2 Results from the online pilot study

Table S2.1 Analysis of affect rating (pilot study)

**Affect Rating**	num df	den df	*F*	*p*	η_p^2
VA of Narr.	1	28	0.67	0.419	0.02
Valence	1	28	125.31	0.000	0.82
Question Type	1	28	0.55	0.465	0.02
VA of Narr. x Valence	1	28	0.98	0.332	0.03
VA of Narr. x Question Type	1	28	0.27	0.610	0.01
Valence x Question Type	1	28	22.63	0.000	0.45
VA of Narr. x Valence x Question Type	1	28	1.08	0.309	0.04

VA of Narr. = Visual Ability of Narrator

Figure S2.1 Mean affect rating (pilot study)

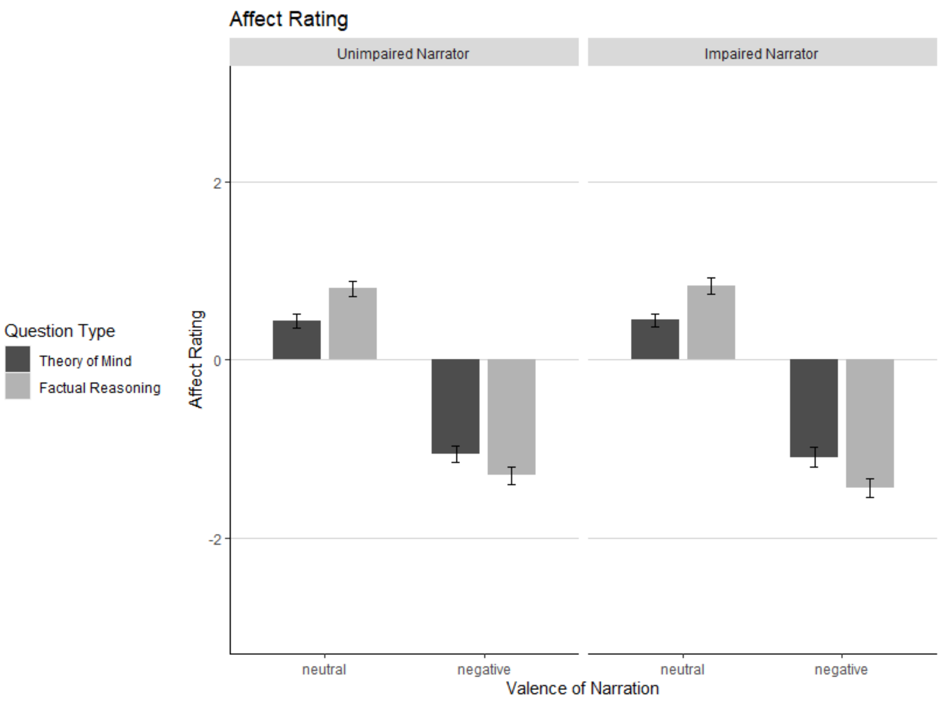

Error bars indicate standard errors.

S2.2 Analysis of accuracy (pilot study)

Accuracy	num df	den df	*F*	*p*	η_p^2
VA of Narr.	1	28	58.31	0.000	0.68
Valence	1	28	0.41	0.529	0.01
Question Type	1	28	22.66	0.000	0.45
VA of Narr. x Valence	1	28	0.08	0.781	0.00
VA of Narr. x Question Type	1	28	8.40	0.007	0.23
Valence x Question Type	1	28	6.51	0.016	0.19
VA of Narr. x Valence x Question Type	1	28	6.85	0.014	0.20

VA of Narr. = Visual Ability of Narrator

Figure S2.2 Mean accuracy (pilot)

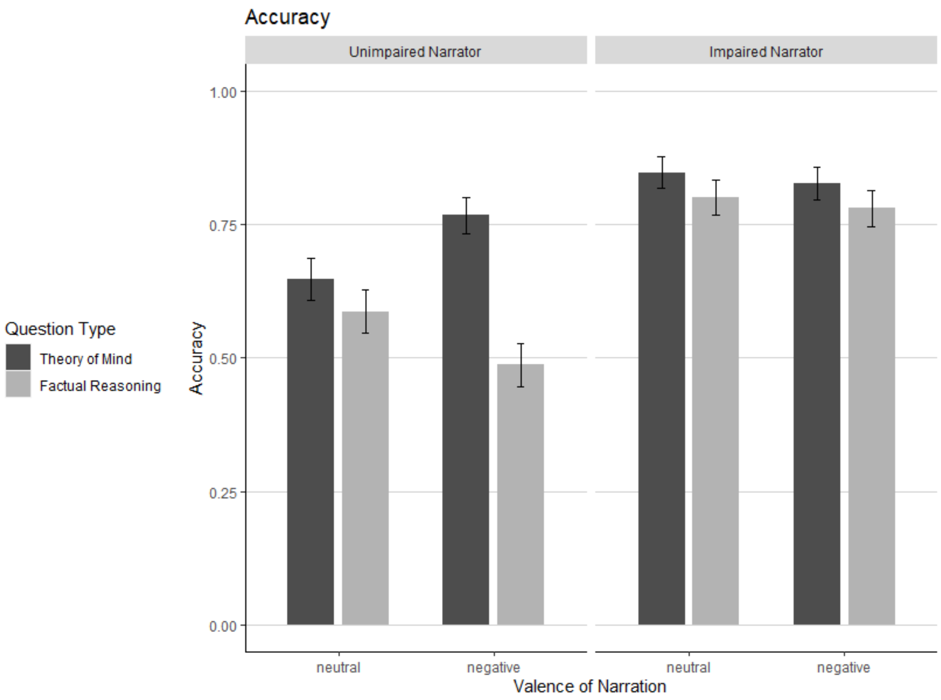

Error bars indicate standard errors.

S3 Main analyses excluding participants who correctly guessed the study aim

Table S3.1 Analysis of affect rating including (left) and excluding (right) participants who correctly guessed the study aim

	Complete Sample					Reduced Sample				
**Affect Rating**	num df	den df	*F*	*p*	η_p^2	num df	den df	*F*	*p*	η_p^2
VA of Part.	1	54	0.93	0.340	0.02	1	49	1.60	0.212	0.03
VA of Narr.	1	54	0.00	0.977	0.00	1	49	0.04	0.837	0.00
VA of Part. x VA of Narr.	1	54	0.11	0.741	0.00	1	49	0.43	0.513	0.01
Valence	1	54	259.89	0.000	0.83	1	49	235.94	0.000	0.83
VA of Part. x Valence	1	54	0.12	0.733	0.00	1	49	0.15	0.702	0.00
Question Type	1	54	11.68	0.001	0.18	1	49	13.95	0.000	0.22
VA of Part. x Question Type	1	54	0.70	0.405	0.01	1	49	0.09	0.76	0.00
VA of Narr. x Valence	1	54	0.75	0.392	0.01	1	49	0.40	0.528	0.01
VA of Part. x VA of Narr. x Valence	1	54	0.05	0.822	0.00	1	49	0.00	0.993	0.00
VA of Narr. x Question Type	1	54	8.63	0.005	0.14	1	49	9.06	0.004	0.16
VA of Part. x VA of Narr. x Question Type	1	54	0.32	0.576	0.01	1	49	0.47	0.495	0.01
Valence x Question Type	1	54	67.85	0.000	0.56	1	49	66.91	0.000	0.58
VA of Part. x Valence x Question Type	1	54	0.31	0.580	0.01	1	49	0.83	0.367	0.02
VA of Narr. x Valence x Question Type	1	54	15.90	0.000	0.23	1	49	16.99	0.000	0.26
VA of Part. x VA of Narr. x Valence x Question Type	1	54	6.39	0.014	0.11	1	49	4.60	0.037	0.09

VA of Part. = Visual Ability of Participant; VA of Narr. = Visual Ability of Narrator

Figure S3.1 Mean affect rating including (upper plot) and excluding (lower plot) participants who correctly guessed the study aim

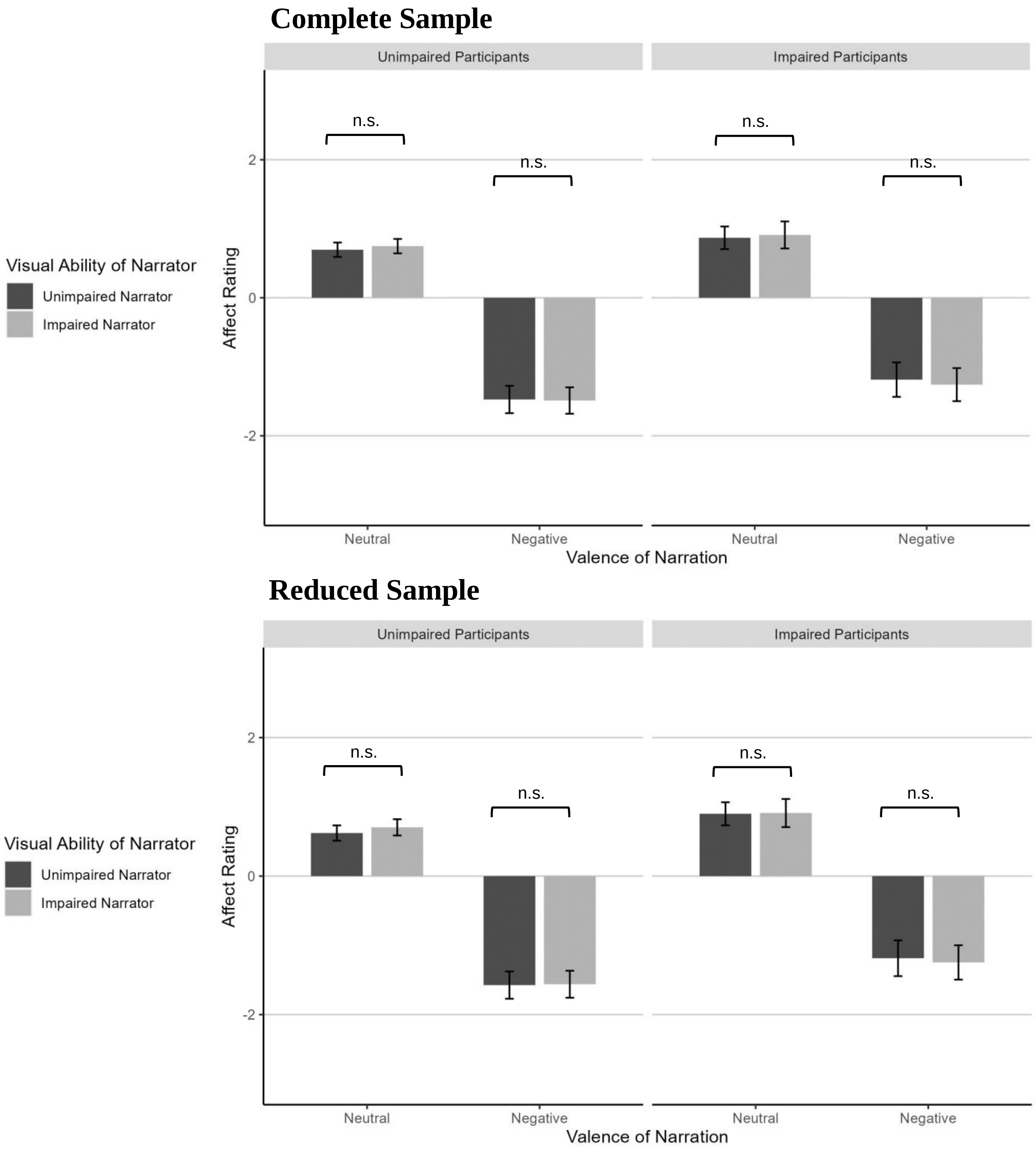

Error bars indicate standard errors. Horizontal brackets indicate pairwise comparisons: n.s.: >= .05. *: $p < .05$. **: $p < .01$. ***: $p < .001$.

Table S3.2 Analysis of accuracy including (left) and excluding (right) participants who correctly guessed the study aim

	Complete Sample					Reduced Sample				
**Accuracy**	num df	den df	*F*	*p*	η_p^2	num df	den df	*F*	*p*	η_p^2
VA of Part.	1	54	4.80	0.033	0.08	1	49	1.93	0.171	0.04
VA of Narr.	1	54	37.73	0.000	0.41	1	49	6.39	0.015	0.12
VA of Part. x VA of Narr.	1	54	3.17	0.081	0.06	1	49	0.38	0.541	0.01
Valence	1	54	1.12	0.295	0.02	1	49	32.37	0.000	0.40
VA of Part. x Valence	1	54	0.10	0.752	0.00	1	49	2.12	0.152	0.04
Question Type	1	54	7.18	0.010	0.12	1	49	0.88	0.352	0.02
VA of Part. x Question Type	1	54	0.21	0.646	0.00	1	49	0.35	0.558	0.01
VA of Narr. x Valence	1	54	7.26	0.009	0.12	1	49	5.63	0.022	0.10
VA of Part. x VA of Narr. x Valence	1	54	9.14	0.004	0.14	1	49	1.60	0.212	0.03
VA of Narr. x Question Type	1	54	6.58	0.013	0.11	1	49	7.92	0.007	0.14
VA of Part. x VA of Narr. x Question Type	1	54	1.68	0.200	0.03	1	49	0.58	0.451	0.01
Valence x Question Type	1	54	11.99	0.001	0.18	1	49	8.42	0.006	0.15
VA of Part. x Valence x Question Type	1	54	0.22	0.644	0.00	1	49	7.72	0.008	0.14
VA of Narr. x Valence x Question Type	1	54	5.52	0.023	0.09	1	49	5.24	0.026	0.10
VA of Part. x VA of Narr. x Valence x Question Type	1	54	0.01	0.942	0.00	1	49	0.00	0.966	0.00

VA of Part. = Visual Ability of Participant; VA of Narr. = Visual Ability of Narrator

Figure S3.2 Mean accuracy including (upper plot) and excluding (lower plot) participants who correctly guessed the study aim

Complete Sample

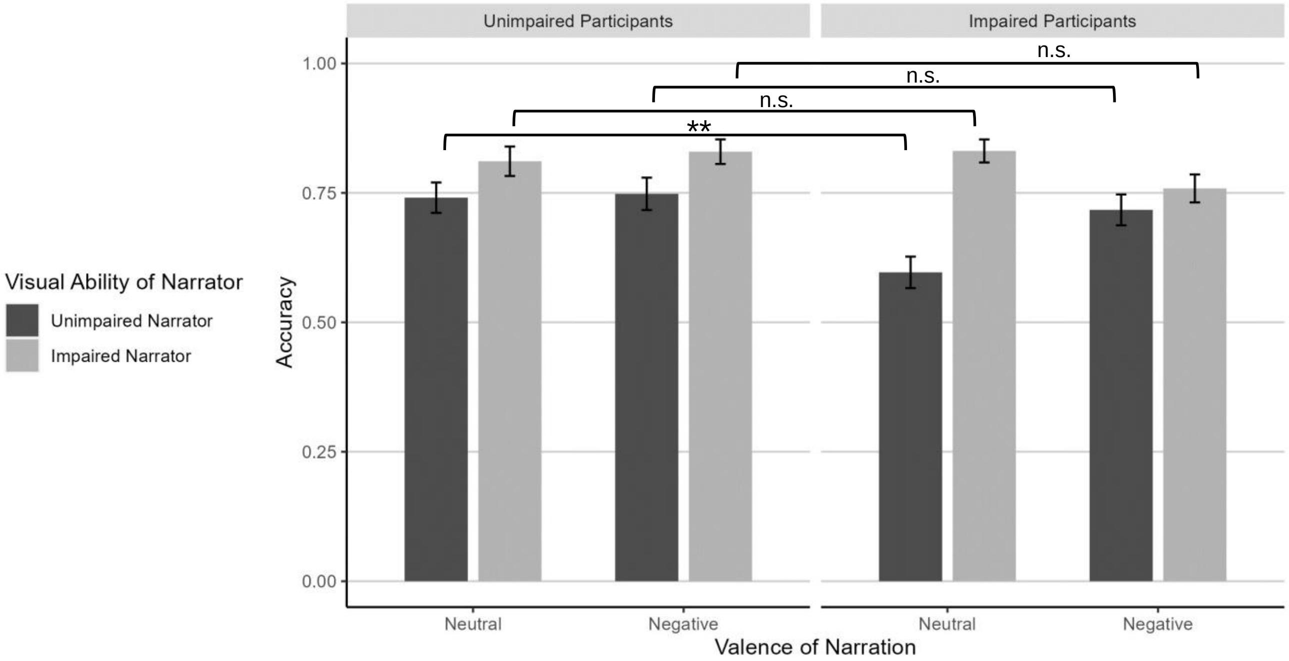

Reduced Sample

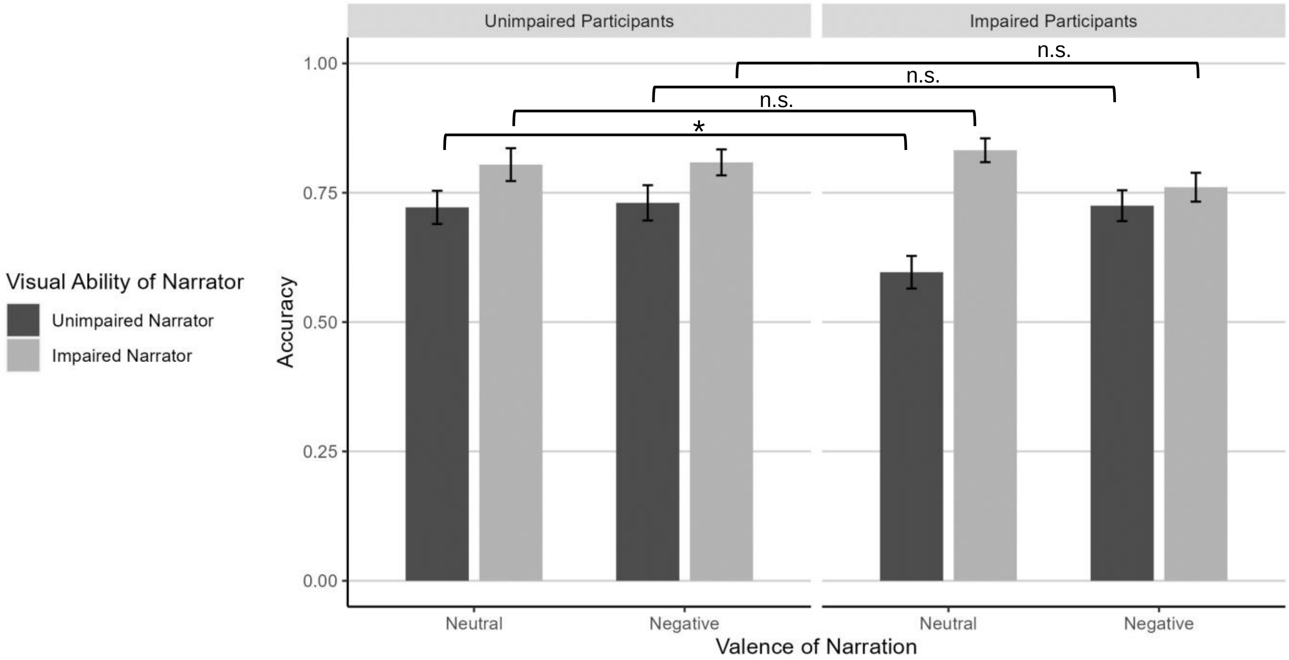

Error bars indicate standard errors. Horizontal brackets indicate pairwise comparisons: n.s.: >= .05. *: $p < .05$. **: $p < .01$. ***: $p < .001$.

S4 Main analyses including only participants with congenital/acquired impairment in the visually impaired subsample

The participant distribution in the visually impaired subsample was not evenly split between those with congenital and acquired visual impairments. Specifically, the majority, nearly two-thirds (19 out of 29), of our visually impaired subgroup had been impaired since birth. Bearing this in mind, our analyses did not indicate significant differences between the groups in their affect ratings (both overall and only for negative narrations, *t*s < 1). Likewise, we did not find overall differences for accuracy, $t < 1$, although participants with acquired visual impairment answered questions with slightly lower accuracy than their counterparts with congenital impairment (71% vs. 74%). Interestingly, however, a significant difference emerged between the groups regarding reported familiarity for narrations by visually unimpaired narrators, $t(16.5) = 2.42$, $p = .028$. On average, participants with acquired impairments rated their familiarity with these narrations higher than participants with congenital impairments, possibly reflecting experiences predating their visual impairment. No significant difference was observed in familiarity ratings regarding visually impaired narrators, $p = .254$.

We then conducted a reanalysis of our main comparisons for Affect Rating (see Table and Plot S4.1) and Response Accuracy (see Table and Plot S4.2), comparing only congenitally impaired with visually unimpaired participants (see next section i). Similar analyses were also conducted for participants with acquired impairment (vs. unimpaired participants) (see next section ii). However, given that this latter group consisted of only 10 participants, we are cautious about interpreting these results. The small sample size likely contributes to non-significant effects, and individual participants within this group could potentially wield significant influence on the overall findings.

(i) Limiting the analysis to participants with congenital visual impairment did not alter the pattern of results relevant to our hypotheses. Specifically, for the dependent variable Affect Rating, we did not observe a significant interaction between Visual Ability of Participant, Visual Ability of Narrator and Valence, $p = .416$, suggesting that empathic responses did not differ based on the match or mismatch of visual abilities. For the dependent variable Response Accuracy, mirroring the main analysis, there was no interaction between Visual Ability of Participant and Visual Ability of Narrator, $p = .132$, but a significant three-way

interaction between Visual Ability of Participant, Visual Ability of Narrator and Valence, $F(1,44) = 12.35$, $p = .001$, suggesting reduced accuracy specifically for neutral narrations by visually unimpaired narrators.

(ii) Restricting the visually impaired subgroup to participants with acquired visual impairments did not result in any significant interactions involving the factors Visual Ability of Participant and Visual Ability of Narrator relevant to our hypotheses for both Affect Rating and Response Accuracy, ps > .169, possibly due to the small power. We observed a marginally significant main effect of Visual Ability of Participant for Response Accuracy, $p = .054$, suggesting lower overall accuracy among participants with acquired visual impairment (compared to visually unimpaired participants). However, we want to underscore again that the sample size of participants with acquired impairment is too small to draw any reliable conclusions.

Table S4.1 Analysis of affect rating including only participants with congenital impairment (left) and acquired impairment (right) in the visually impaired subsample

	Unimpaired vs. Congenitally Impaired Participants					Unimpaired vs. Acquired Impaired Participants				
**Affect Rating**	num df	den df	*F*	*p*	η_p^2	num df	den df	*F*	*p*	η_p^2
VA of Part.	1	44	0.29	0.596	0.01	1	35	1.71	0.200	0.05
VA of Narr.	1	44	0.37	0.547	0.01	1	35	0.53	0.472	0.01
VA of Part. x VA of Narr.	1	44	0.05	0.827	0.00	1	35	0.99	0.327	0.03
Valence	1	44	222.68	0.000	0.84	1	35	145.71	0.000	0.81
VA of Part. x Valence	1	44	0.00	0.989	0.00	1	35	0.64	0.430	0.02
Question Type	1	44	8.12	0.007	0.16	1	35	7.06	0.012	0.17
VA of Part. x Question Type	1	44	0.45	0.506	0.01	1	35	0.49	0.487	0.01
VA of Narr. x Valence	1	44	2.14	0.150	0.05	1	35	0.04	0.840	0.00
VA of Part. x VA of Narr. x Valence	1	44	0.67	0.416	0.02	1	35	0.51	0.481	0.01
VA of Narr. x Question Type	1	44	4.31	0.044	0.09	1	35	9.35	0.004	0.21
VA of Part. x VA of Narr. x Question Type	1	44	0.85	0.363	0.02	1	35	0.10	0.750	0.00
Valence x Question Type	1	44	81.45	0.000	0.65	1	35	24.36	0.000	0.41
VA of Part. x Valence x Question Type	1	44	0.01	0.944	0.00	1	35	1.50	0.229	0.04
VA of Narr. x Valence x Question Type	1	44	11.45	0.002	0.21	1	35	11.21	0.002	0.24
VA of Part. x VA of Narr. x Valence x Question Type	1	44	3.90	0.055	0.08	1	35	5.53	0.024	0.14

VA of Part. = Visual Ability of Participant; VA of Narr. = Visual Ability of Narrator

Figure S4.1 Mean affect rating including only participants with congenital impairment (upper plot) and acquired impairment (lower plot) in the visually impaired subsample

Unimpaired vs. congenitally impaired participants

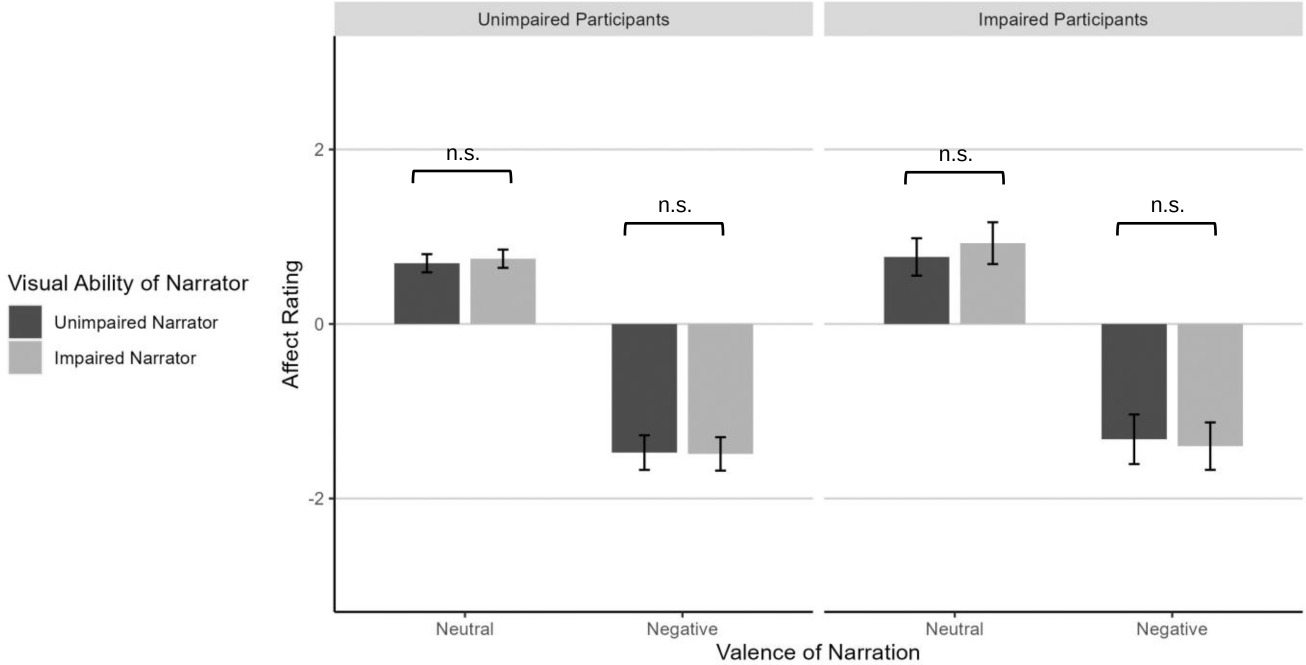

Unimpaired vs. acquired impaired participants

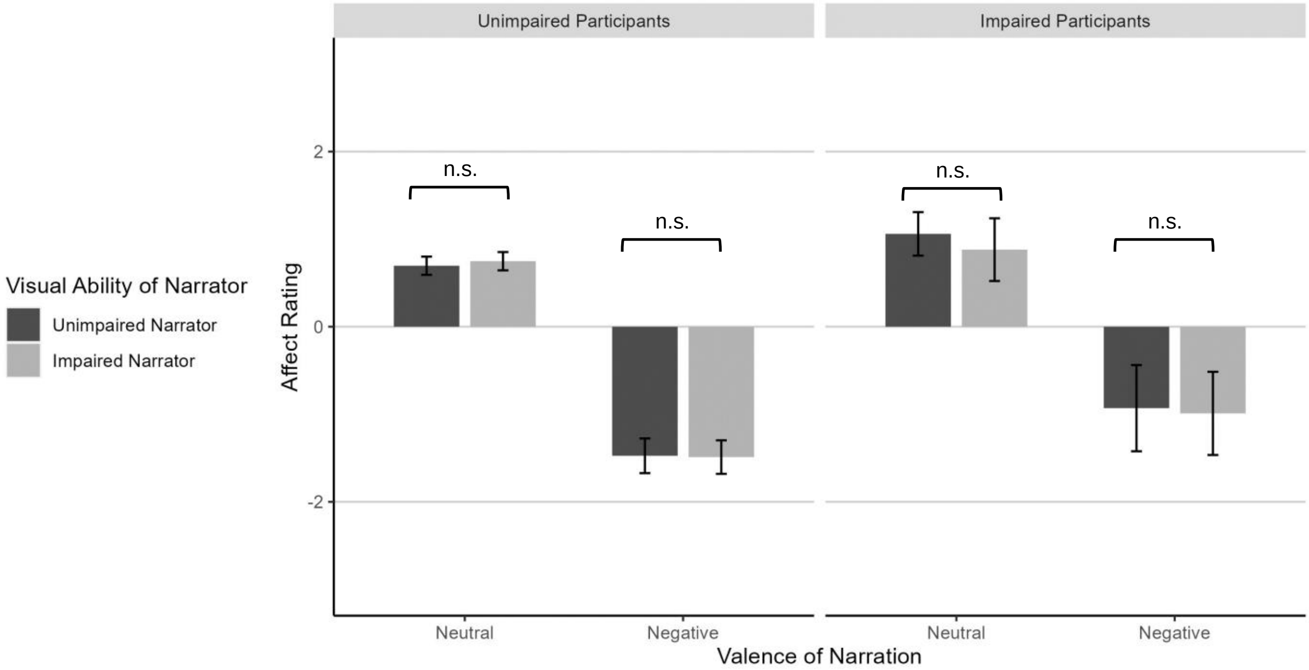

Error bars indicate standard errors. Horizontal brackets indicate pairwise comparisons: n.s.: >= .05. *: $p < .05$. **: $p < .01$. ***: $p < .001$.

Table S4.2 Analysis of accuracy including only participants with congenital impairment (left) and acquired impairment (right) in the visually impaired subsample

	Unimpaired vs. Congenitally Impaired Participants					Unimpaired vs. Acquired Impaired Participants				
**Accuracy**	num df	den df	*F*	*p*	η_p^2	num df	den df	*F*	*p*	η_p^2
VA of Part.	1	44	2.62	0.113	0.06	1	35	3.98	0.054	0.10
VA of Narr.	1	44	30.64	0.000	0.41	1	35	20.14	0.000	0.37
VA of Part. x VA of Narr.	1	44	2.36	0.132	0.05	1	35	1.97	0.169	0.05
Valence	1	44	3.67	0.062	0.08	1	35	0.20	0.657	0.01
VA of Part. x Valence	1	44	1.41	0.241	0.03	1	35	0.95	0.336	0.03
Question Type	1	44	7.33	0.010	0.14	1	35	2.77	0.105	0.07
VA of Part. x Question Type	1	44	0.06	0.806	0.00	1	35	0.37	0.548	0.01
VA of Narr. x Valence	1	44	10.32	0.002	0.19	1	35	0.73	0.399	0.02
VA of Part. x VA of Narr. x Valence	1	44	12.35	0.001	0.22	1	35	1.20	0.281	0.03
VA of Narr. x Question Type	1	44	4.91	0.032	0.10	1	35	3.64	0.065	0.09
VA of Part. x VA of Narr. x Question Type	1	44	1.21	0.277	0.03	1	35	0.99	0.325	0.03
Valence x Question Type	1	44	14.87	0.000	0.25	1	35	1.73	0.197	0.05
VA of Part. x Valence x Question Type	1	44	1.31	0.259	0.03	1	35	0.50	0.484	0.01
VA of Narr. x Valence x Question Type	1	44	9.97	0.003	0.18	1	35	0.11	0.739	0.00
VA of Part. x VA of Narr. x Valence x Question Type	1	44	1.05	0.311	0.02	1	35	2.38	0.132	0.06

VA of Part. = Visual Ability of Participant; VA of Narr. = Visual Ability of Narrator

Figure S4.2 Mean accuracy including only participants with congenital impairment (upper plot) and acquired impairment (lower plot) in the visually impaired subsample

Unimpaired vs. congenitally impaired participants

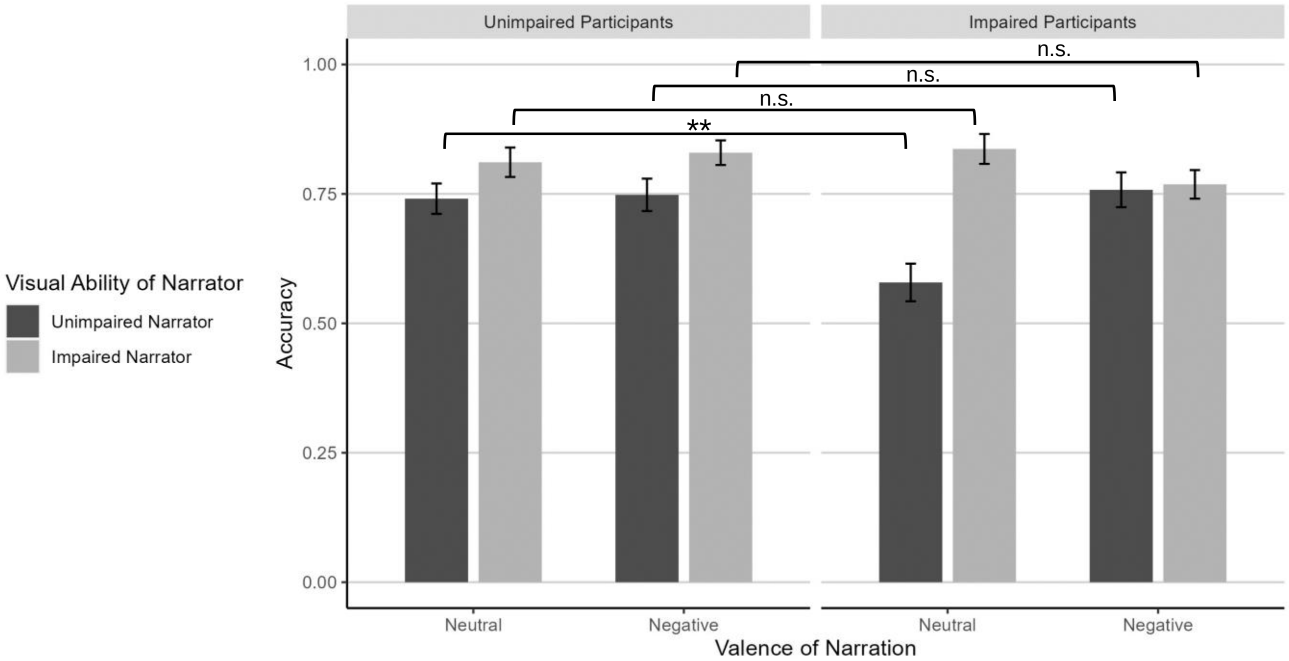

Unimpaired vs. acquired impaired participants

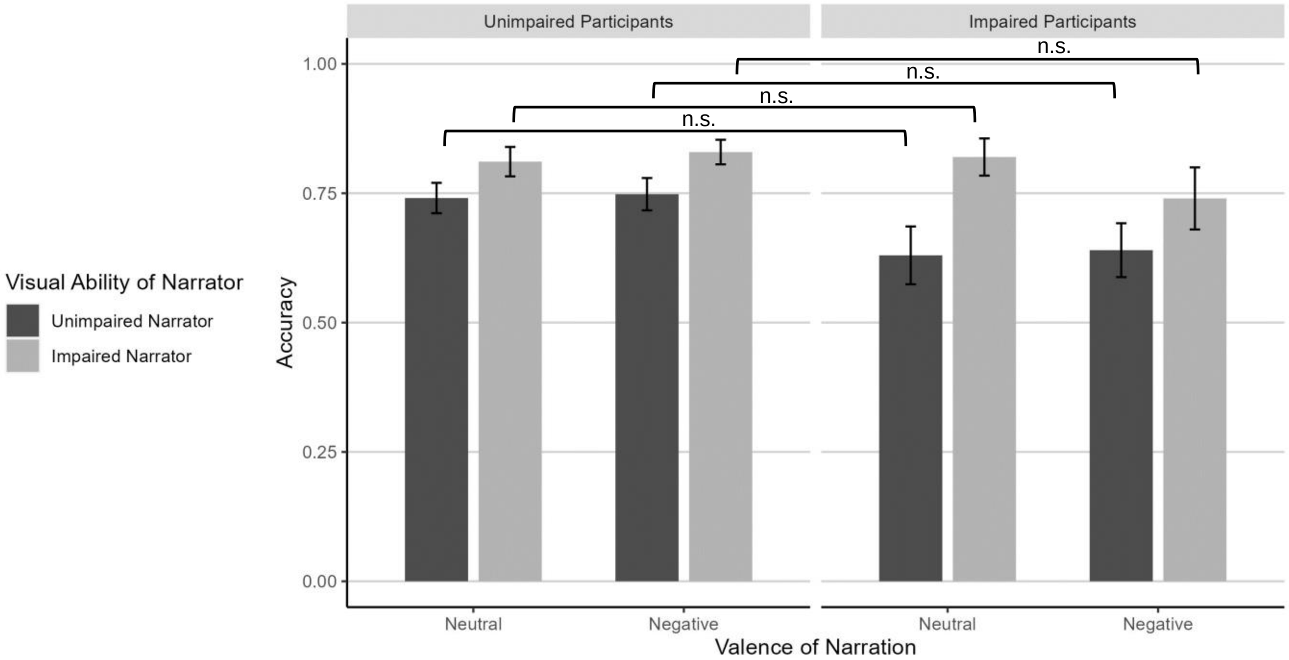

Error bars indicate standard errors. Horizontal brackets indicate pairwise comparisons: n.s.: >= .05. *: $p < .05$. **: $p < .01$. ***: $p < .001$.

S5 Exploratory analyses regarding the importance of visual abilities in the narrations

The narrations we employed as stimuli in our study were heterogeneous regarding whether the narrator's visual abilities played a central role in the narrative or were (more or less) incidental. This range was partly by design since it was important to us to capture a nuanced and comprehensive representation of the everyday lives of visually impaired individuals. We felt that focusing on visual impairment as the central theme in every newly generated narration might compromise ecological validity as this restriction neither matched the pre-existing sets of narrations by visually unimpaired narrators nor the life experiences shared with us by visually impaired people. In order to avoid creating one-dimensional narrators defined solely by their impairment – who might elicit compassion at the beginning, but not evoke realistic reactions – we crafted the narrations in a way that would make it possible to correctly identify the visual abilities of the narrator, but that did not reduce the narrators to this singular trait.

To explore whether this heterogeneity influenced our results, we classified our narrations into three distinct categories:

- Narrations in which the narrator's visual abilities are **incidental**, meaning that the described events could be experienced in the same way by a person with different visual abilities save for minor and inconsequential changes (e.g., going to the cinema and mentioning the audio-description).
- Narrations for whose core the narrator's visual abilities are **influential** in the sense that the described experiences are either much more probable/typical for a certain group (e.g., working as a barkeeper) or that the visual abilities affect the emotional impact of the experience (e.g., heightened feelings of helplessness due to an impairment).
- Narrations for whose core the narrator's visual abilities are **essential**, meaning that the experience virtually cannot happen to a person with different visual abilities (e.g., describing an accident causing a loss of vision).

Upon reviewing all 80 narrations, the following aspects emerged, which lead us to classify narrations as either influential or essential regarding the role of visual abilities:

- Influential role of visual abilities:
  - Visually unimpaired narrators
    - working a job that is unusual for visually impaired individuals
    - engaging in activities that are unusual for visually impaired individuals
    - describing visual impressions as central part of the narration
  - Visually impaired narrators
    - having to rely on assistance to perform everyday tasks
    - experiencing added emotional impact due to impairment
    - encountering impeded communication due to impairment
- Essential role of visual abilities:
  - Visually unimpaired narrators
    - working a job that at its core depends on visual abilities
    - feeling guilty for actions only possible because of unimpaired visual abilities
  - Visually impaired narrators
    - being judged or treated favorably explicitly because of impairment
    - being judged or treated negatively explicitly because of impairment
    - using assistive devices only used by persons with visual impairments
    - recounting the loss of sight and its impact on self or loved ones

To provide a comprehensive overview, here are illustrative examples for each category:

**Role of visual abilities**	**Visually unimpaired narrator**	**Visually impaired narrator**
**Incidental**	It's really not that easy in a shared flat. But Paul has come up with a system... with a list. And we rotate through it. But Eva always forgets to sign in when she's done the cleaning. And then Paul signs in... I think he then thinks that somehow Eva cleans twice...	I recently discovered a new hiking group. Now I can finally pursue my old hobby again. Hmm, and we've already climbed a few peaks! And you always get to know interesting guides there too.
**Influential**	I was trained as a fitness instructor, and I've been working in a gym for	When I told my mother that I'm pregnant, I really hoped she would

	years. I take care of the training plans, but also the nutrition plans and make sure that the customers do their exercises correctly. Well, for the really tough ones I also offer a boot camp from time to time.<br><br>*[working a job that is unusual for visually impaired individuals]*	be happy for me... Can't she understand that, despite my disability, I want to have a baby?... but she just kept pushing, 'come on, it's not too late yet'... I regret so much that I listened to her.<br><br>*[experiencing added emotional impact due to impairment]*
**Essential**	I only saw the woman at the last moment... behind the bus stop. I stopped the bus immediately... But somehow I must have caught her with the mirror. And then she went under the bus…<br><br>*[feeling guilty for actions only possible because of unimpaired visual abilities]*	During the birth of my third child, there were complications... I had terrible pain in my head and eyes – for weeks... (Voice trembling) The doctors explained to me that the pressure had led to damage to the optic nerve...<br><br>*[recounting the loss of sight and its impact on self or loved ones]*

The narrations by visually unimpaired (VU) and visually impaired (VI) narrators fell into the three categories with roughly equal ratios. In a third of the narrations (26, VU: 13, VI: 13) visual abilities played only an incidental role. In 32 of the narrations (VU: 17, VI: 15) visual abilities influenced the core of the narration, while in the remaining 22 narrations (VU: 10, VI: 12) it was essential to the narration. Due to the compilation of narrations each participant was presented with at least 8 and a maximum of 14 narrations in which visual abilities of the narrator played a role we considered essential (incidental: 10-15 narrations; influential: 14-18 narrations).

To explore whether possible effects of matching realities of life were attenuated by the inclusion of narrations in which the narrator’s visual abilities were incidental to the core of the described experience, we re-conducted our main analyses including only narrations in which the visual abilities were classified as influential or essential. The pattern of results remained consistent both for Affect Rating (see Table and Plot S5.1) and Response Accuracy Rating (see Table and Plot S5.2), meaning we observed no significant effect of matching

visual abilities for empathic responses and accuracy, *p*s > .099, and the only notable reduction in response accuracy was found among visually impaired participants listening to narrations by visually unimpaired narrators, interaction Visual Ability of Participant x Visual Ability of Narrator x Valence, $F(1,54) = 17.26$, $p = .001$.

We also conducted further additional analyses, exploring the potential effect of narrations in which visual abilities played a core role, on the empathic process. Placing special emphasis on negative narrations centering around visual abilities, we selected a subset of narrators for whom one or both negative narrations had been classified as “essential”. This criterion applied to 5 visually impaired and 5 visually unimpaired narrators, i.e., half of our sets (see S1 for two examples: Helga and Marita). Reconducting our main analyses for Affect Rating (see Table and Plot S5.1), the results remained virtually unchanged from the full stimulus set (interaction Visual Ability of Participant x Visual Ability of Narrator x Valence: $p = .902$). For the sake of completeness, we also repeated the analysis for Response Accuracy (see Table and Plot S5.2) with this subset of narrators. As in the main analysis, there was no significant interaction between Visual Ability of Participant and Visual Ability of Narrator, $p = .586$, but a significant three-way interaction Visual Ability of Participant x Visual Ability of Narrator x Valence, $F(1,54) = 8.63$, $p = .005$. However, after adjusting for alpha-inflation none of the post-hoc *t*-tests comparing visually unimpaired and impaired participants in the different conditions reached significance.

In conclusion, our exploratory analyses did not yield indications of differences between narrations wherein the narrator's visual abilities were incidental versus those where they were central to the core of the narration.

Table S5.1 Analysis of affect rating including only a subset of narrations with visual abilities at their core (left) and a subset of narrators with visual abilities at their core (right)

	Subset of narrations with visual abilities at their core					Subset of narrators with visual abilities at their core				
**Affect Rating**	num df	den df	*F*	*p*	η_p^2	num df	den df	*F*	*p*	η_p^2
VA of Part.	1	54	0.70	0.408	0.01	1	54	1.23	0.273	0.02
VA of Narr.	1	54	0.00	0.996	0.00	1	54	0.08	0.773	0.00
VA of Part. x VA of Narr.	1	54	0.02	0.890	0.00	1	54	0.21	0.651	0.00
Valence	1	54	274.71	0.000	0.84	1	54	269.50	0.000	0.83
VA of Part. x Valence	1	54	0.27	0.603	0.01	1	54	0.11	0.740	0.00
Question Type	1	54	6.80	0.012	0.11	1	54	1.53	0.222	0.03
VA of Part. x Question Type	1	54	1.29	0.261	0.02	1	54	0.00	0.954	0.00
VA of Narr. x Valence	1	54	0.86	0.358	0.02	1	54	0.06	0.805	0.00
VA of Part. x VA of Narr. x Valence	1	54	0.00	1.000	0.00	1	54	0.02	0.902	0.00
VA of Narr. x Question Type	1	54	8.92	0.004	0.14	1	54	1.39	0.244	0.03
VA of Part. x VA of Narr. x Question Type	1	54	2.13	0.150	0.04	1	54	0.35	0.557	0.01
Valence x Question Type	1	54	38.57	0.000	0.42	1	54	37.93	0.000	0.41
VA of Part. x Valence x Question Type	1	54	0.01	0.921	0.00	1	54	0.05	0.826	0.00
VA of Narr. x Valence x Question Type	1	54	4.43	0.040	0.08	1	54	3.79	0.057	0.07
VA of Part. x VA of Narr. x Valence x Question Type	1	54	3.96	0.052	0.07	1	54	6.07	0.017	0.10

VA of Part. = Visual Ability of Participant; VA of Narr. = Visual Ability of Narrator

Figure S5.1 Mean affect rating including only a subset of narrations with visual abilities at their core (upper plot) and a subset of narrators with visual abilities at their core (lower plot)

Subset of narrations with visual abilities at their core

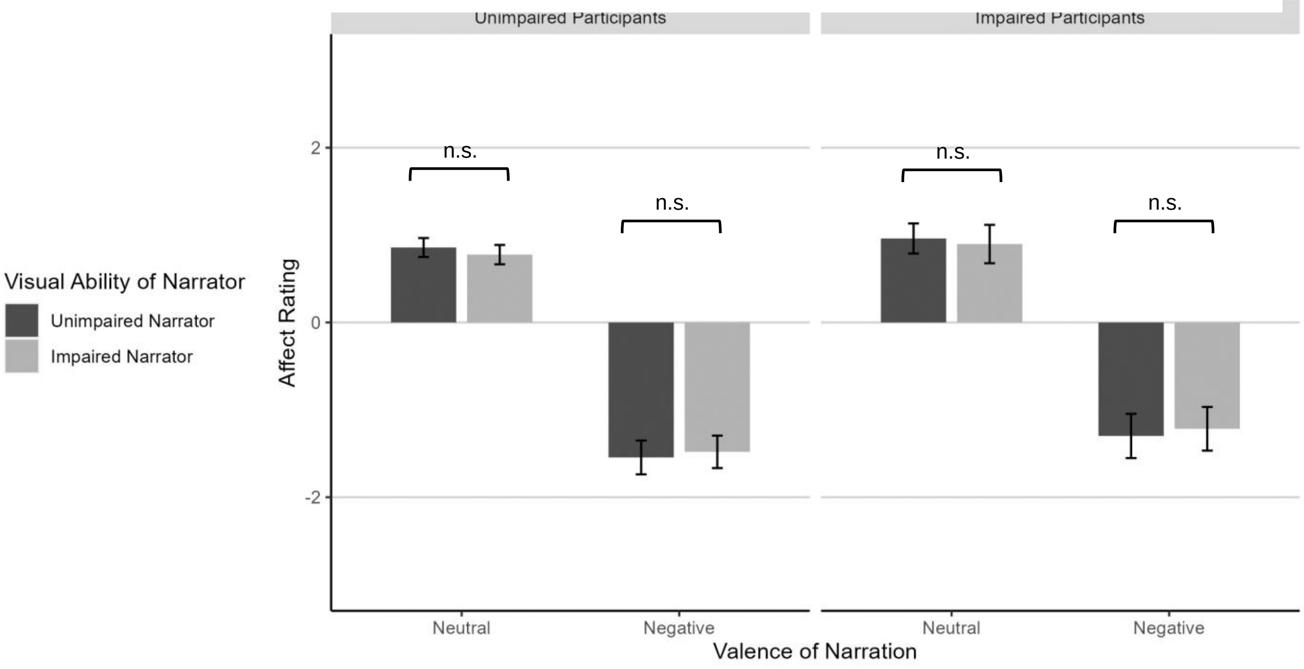

Subset of narrators with visual abilities at their core

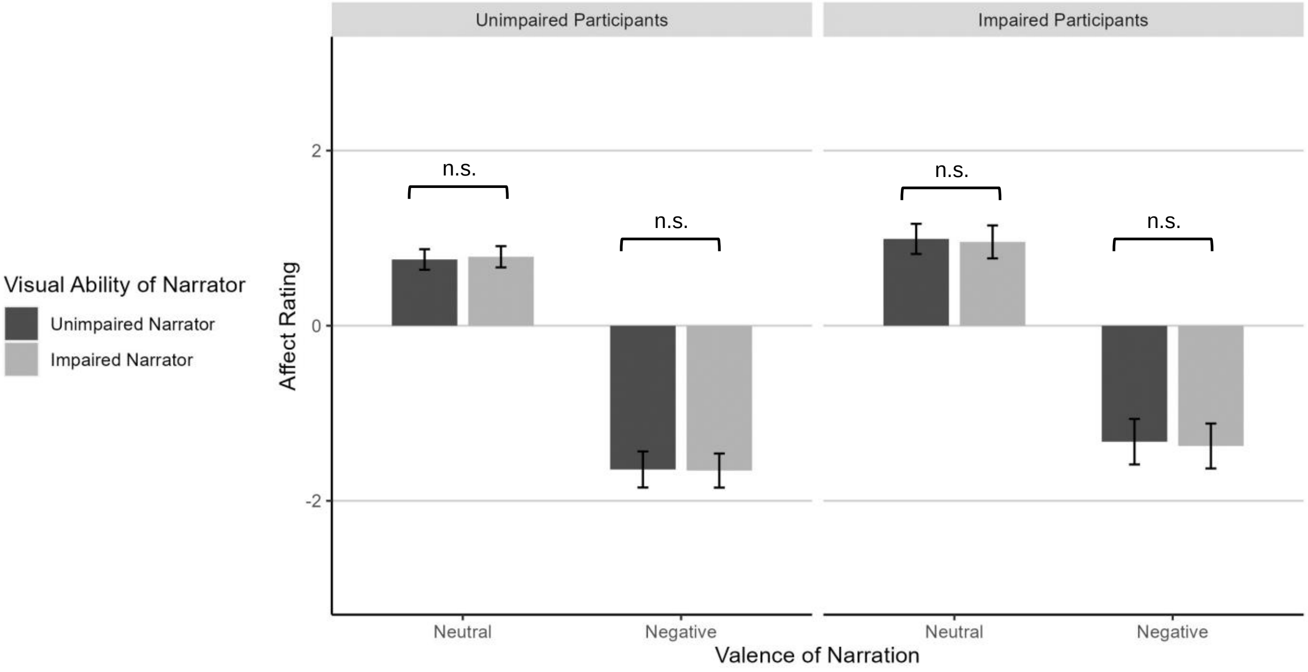

Error bars indicate standard errors. Horizontal brackets indicate pairwise comparisons: n.s.: >= .05. *: $p < .05$. **: $p < .01$. ***: $p < .001$.

Table S5.2 Analysis of accuracy including only a subset of narrations with visual abilities at their core (left) and a subset of narrators with visual abilities at their core

	Subset of narrations with visual abilities at their core					Subset of narrators with visual abilities at their core				
**Accuracy**	num df	den df	*F*	*p*	η_p^2	num df	den df	*F*	*p*	η_p^2
VA of Part.	1	54	2.93	0.093	0.05	1	54	0.63	0.429	0.01
VA of Narr.	1	54	30.73	0.000	0.36	1	54	20.94	0.000	0.28
VA of Part. x VA of Narr.	1	54	2.82	0.099	0.05	1	54	0.30	0.586	0.01
Valence	1	54	1.33	0.253	0.02	1	54	2.43	0.125	0.04
VA of Part. x Valence	1	54	0.00	0.985	0.00	1	54	0.50	0.481	0.01
Question Type	1	54	9.31	0.004	0.15	1	54	0.02	0.895	0.00
VA of Part. x Question Type	1	54	0.10	0.754	0.00	1	54	1.72	0.195	0.03
VA of Narr. x Valence	1	54	13.43	0.001	0.20	1	54	0.03	0.872	0.00
VA of Part. x VA of Narr. x Valence	1	54	17.26	0.000	0.24	1	54	8.63	0.005	0.14
VA of Narr. x Question Type	1	54	3.01	0.088	0.05	1	54	4.11	0.048	0.07
VA of Part. x VA of Narr. x Question Type	1	54	0.04	0.836	0.00	1	54	0.07	0.797	0.00
Valence x Question Type	1	54	10.75	0.002	0.17	1	54	4.54	0.038	0.08
VA of Part. x Valence x Question Type	1	54	1.49	0.228	0.03	1	54	0.15	0.700	0.00
VA of Narr. x Valence x Question Type	1	54	0.01	0.933	0.00	1	54	6.95	0.011	0.11
VA of Part. x VA of Narr. x Valence x Question Type	1	54	0.61	0.436	0.01	1	54	0.15	0.697	0.00

VA of Part. = Visual Ability of Participant; VA of Narr. = Visual Ability of Narrator

Figure S5.2 Mean accuracy including only a subset of narrations with visual abilities at their core (upper plot) and a subset of narrators with visual abilities at their core (lower plot)

Subset of narrations with visual abilities at their core

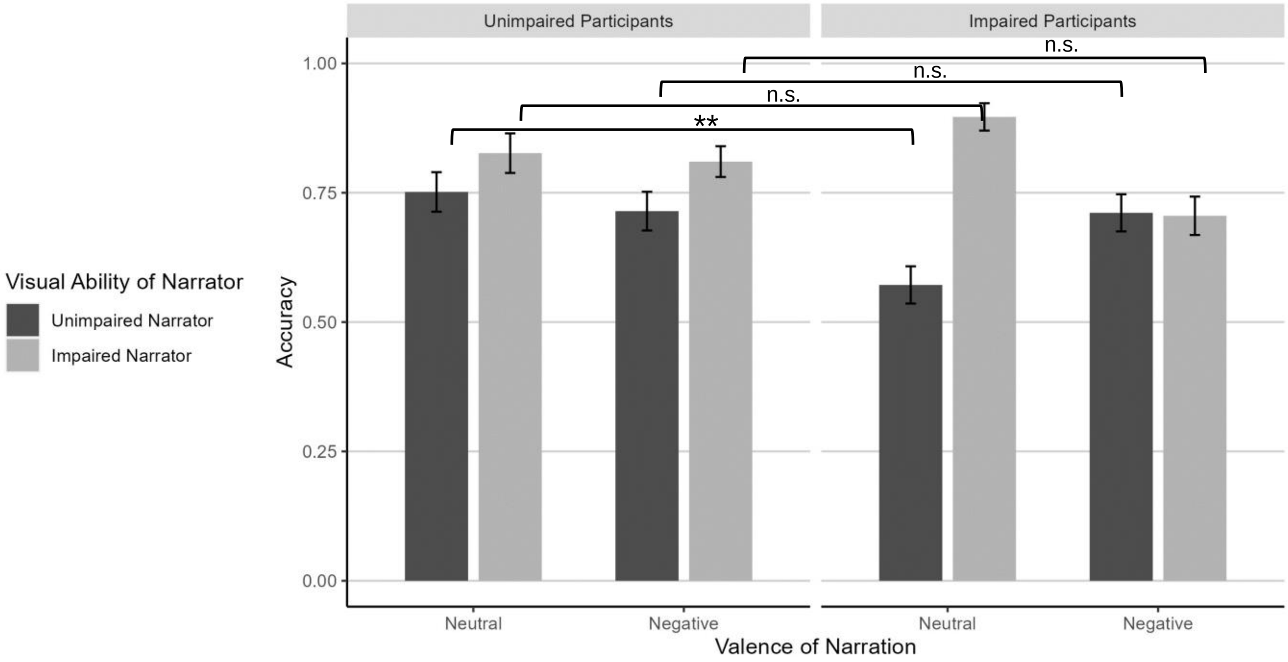

Subset of narrators with visual abilities at their core

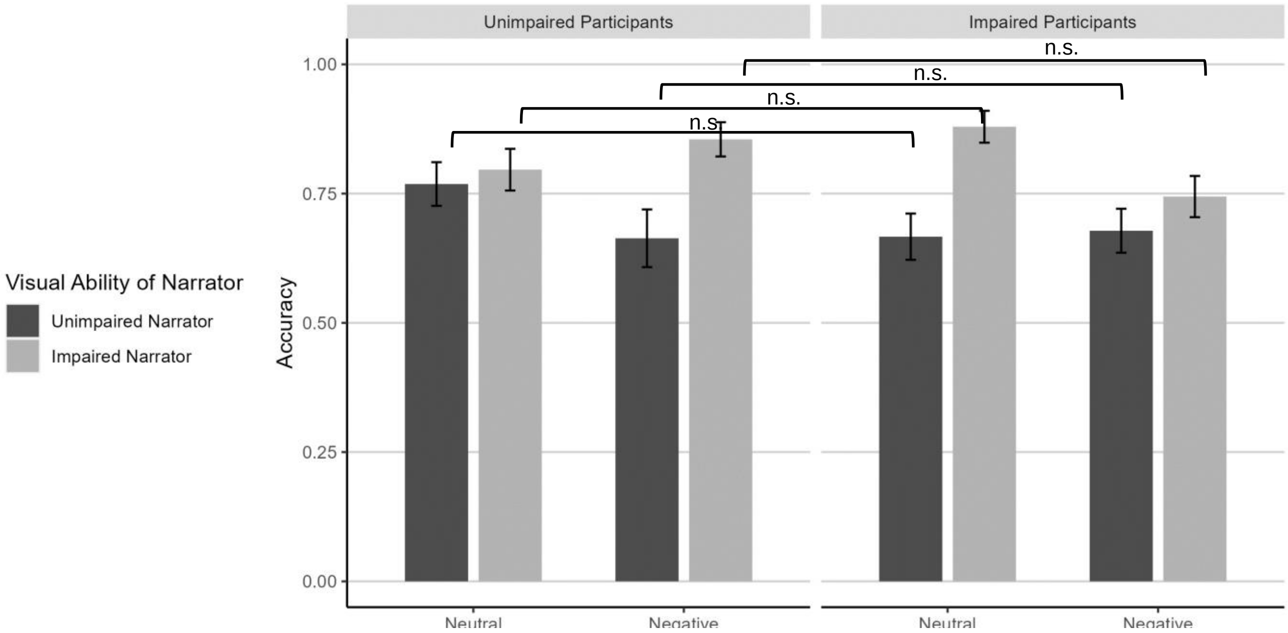

Error bars indicate standard errors. Horizontal brackets indicate pairwise comparisons: n.s.: >= .05. *: $p < .05$. **: $p < .01$. ***: $p < .001$.
